# A ubiquitin-specific, proximity-based labeling approach for the identification of ubiquitin ligase substrates

**DOI:** 10.1126/sciadv.adp3000

**Published:** 2024-08-09

**Authors:** Urbi Mukhopadhyay, Sophie Levantovsky, Teresa Maria Carusone, Sarah Gharbi, Frank Stein, Christian Behrends, Sagar Bhogaraju

**Affiliations:** ^1^ European Molecular Biology Laboratory, 71 avenue des Martyrs, 38042 Grenoble, France.; ^2^ Munich Cluster for Systems Neurology, Medical Faculty, Ludwig-Maximilians-Universität München, Munich, Germany.; ^3^ Proteomics Core Facility, European Molecular Biology Laboratory, Heidelberg, Germany.

## Abstract

Over 600 E3 ligases in humans execute ubiquitination of specific target proteins in a spatiotemporal manner to elicit desired signaling effects. Here, we developed a ubiquitin-specific proximity-based labeling method to selectively biotinylate substrates of a given ubiquitin ligase. By fusing the biotin ligase BirA and an Avi-tag variant to the candidate E3 ligase and ubiquitin, respectively, we were able to specifically enrich bona fide substrates of a ligase using a one-step streptavidin pulldown under denaturing conditions. We applied our method, which we named Ub-POD, to the really interesting new gene (RING) E3 ligase RAD18 and identified proliferating cell nuclear antigen and several other critical players in the DNA damage repair pathway. Furthermore, we successfully applied Ub-POD to the RING ubiquitin ligase tumor necrosis factor receptor–associated factor 6 and a U-box–type E3 ubiquitin ligase carboxyl terminus of Hsc70-interacting protein. We anticipate that our method could be widely adapted to all classes of ubiquitin ligases to identify substrates.

## Teaser

A method is developed to identify the substrates of ubiquitin ligases.

## INTRODUCTION

Similar to phosphorylation, ubiquitination of proteins has been found to have a role in virtually every cellular process in which it was investigated (*1*). Ubiquitination of substrates is achieved by three enzymes, E1, E2, and E3, acting in a cascade reaction and involves the covalent attachment of the terminal carboxyl group of ubiquitin (Ub), a 76–amino acid–long polypeptide, to substrate lysine through an isopeptide bond. The human genome encodes two E1s or Ub-activating enzymes, Uba1 and Uba6, that first adenylate the terminal carboxyl group of Ub using an adenosine triphosphate molecule, followed by the formation of a thioester linkage between Ub C terminus and the catalytic cysteine of E1. There are ~40 E2s or Ub-conjugating enzymes that bind to E1 and catalyze a transthioesterification reaction, resulting in an E2~Ub thioester conjugate. It is estimated that humans encode for >600 E3 Ub ligases that confer specificity and functionality to the Ub cascade by targeted ubiquitination of thousands of cellular substrates (*1*). E3s bind the E2~Ub conjugate and the substrate to facilitate the transfer of Ub to target lysines of the substrate. The really interesting new gene (RING) family of E3 ligases comprise most of the E3s in the cell, with an estimated ~600 members (*2*). RING E3 ligases contain a conserved Zn^2+^-binding RING domain and other auxiliary domains that bind E2~Ub and substrates, respectively. The RING domain spans ~70 residues and adopts a conserved α/β topology. In RING E3–catalyzed ubiquitination, Ub is transferred directly from the catalytic cysteine of E2 to the substrate lysine residues. U-box E3 ligases also contain a RING-like catalytic domain that lacks the Zn^2+^-binding site and operate as facilitators of ubiquitination, akin to the RING E3 ligases (*3*). Homologous to E6–acceptor peptide (AP) carboxyl terminus (HECT) family comprises another major class of E3 ligase, with ~20 members in the human genome. HECT E3 ligases contain a conserved bilobal HECT domain at the C terminus of the protein. The C-lobe of the HECT domain contains an invariant cysteine that accepts Ub from E2~Ub, before transferring Ub to substrates. RBR (RING-between-RING) type Ub ligases have two RING domains, one of which contains a catalytic cysteine that becomes transiently thioester linked to Ub, before substrate ubiquitination (*4*).

Because E3 ligases are the concluding and decisive actors of all Ub signaling events, substrate identification of a given Ub ligase is critical for gaining insights into its cellular and physiological roles. Traditional approaches of identifying protein-protein interactions, such as yeast two-hybrid and immunoprecipitation-coupled proteomics, have provided valuable resources of potential substrates and the cellular pathways a given E3 ligase engages with (*5*). However, in most cases, E3-substrate interactions are transient and difficult to capture by these conventional methods. Many alternate methods have been developed to identify E3 ligase substrates and are reviewed elsewhere (*6*, *7*) in detail. A few such notable approaches are introduced here to provide context for the substrate identification method developed by us. One of the most powerful methods for the identification of E3 ligase substrates uses an antibody that specifically recognizes a diGly remnant that is characteristic of tryptic digested ubiquitinated substrates (*8*). Using quantitative comparison of diGly peptides from cells expressing wild-type (WT) or activity-deficient mutant version of E3 ligases, one can identify the substrates and also the target lysines that are modified. Global protein stability (GPS) profiling uses degradation profiles of potential substrates fused to EGFP, coupled with the genetic perturbation of the E3 ligase of interest to identify substrates (*9*, *10*). Another approach, Ubiquitin-Activated Interaction Traps (UBAITS), uses recombinant fusing of Ub to the E3 ligase of interest to covalently trap the ligase and its cognate substrates, as well as other interacting proteins (*11*). Targets for ubiquitin ligases identified by proteomics (TULIP) and TULIP2 methodologies build on the UBAITS principle and use His-tagged Ub-E3 ligase fusion, allowing purification of the E3 ligase–substrate adduct in denaturing conditions (*12*, *13*). E2-dID uses biotin-labeled Ub-E2 conjugates, plus WT or activity-deficient mutant E3 ligase of interest, added to the whole-cell lysate in vitro to identify potential substrates (*14*). An orthogonal Ub transfer (OUT) approach has also been developed using engineered E1 and E2 enzymes and E3 ligase of interest that do not cross-react with the endogenous Ub system, thus aiming to specifically target the substrates of the engineered Ub ligase of interest (*15*, *16*). Other notable methods include using a microarray of purified human proteins in an in vitro assay (*17*) and a NEDDylator approach, which uses Nedd8-E2 enzyme fused to the E3 ligase of interest (*18*). While no particular method provides a silver bullet solution for the identification of E3 ligase substrates, each method has been proven successful with a specific or a set of ligases, and together, these methods provide an important toolbox in Ub biology research.

Here, we developed an approach to selectively and robustly label the substrates of a given E3 Ub ligase directly in cells. Our approach relies on the conserved but transient intermediate complexes that occur between E2~Ub and the E3 ligase in the case of RING E3 ligases. On the basis of the structural information of E2~Ub–RING E3 (*19*, *20*) complexes, we tagged the ligases of our interest with the *Escherichia coli* (*E. coli*) biotin ligase BirA enzyme and Ub with an AP tag. We cotransfected these constructs in cells, resulting in the proximity- and orientation-dependent tagging of Ub (Ub-POD) during the BirA-E3–mediated ubiquitination, leading to biotin-labeled substrates. We first tested this strategy using two unrelated human RING E3 ligases, RAD18 and tumor necrosis factor (TNF) receptor–associated factor 6 (TRAF6), which are involved in ultraviolet (UV)–induced translesion DNA synthesis and nuclear factor κB (NF-κB) signaling, respectively. We also localized ubiquitination mediated by RAD18 precisely in cells in a Ub-POD experiment using streptavidin immunofluorescence. Next, we successfully used Ub-POD to a U-box domain containing ligase, carboxyl terminus of Hsc70-interacting protein (CHIP)/STIP1 homology and U-box–containing protein 1 (STUB1), which is involved in the protein quality control machinery by recognizing misfolded proteins and targeting them for degradation. Last, we delineate the principles and parameters of Ub-POD to facilitate other Ub researchers to adopt Ub-POD and identify the substrates of their favorite ligases.

## RESULTS

### Concept of Ub-POD: A Ub-specific, proximity- and orientation-dependent labeling approach for identifying E3 Ub ligase substrates

**Fig. 1.**
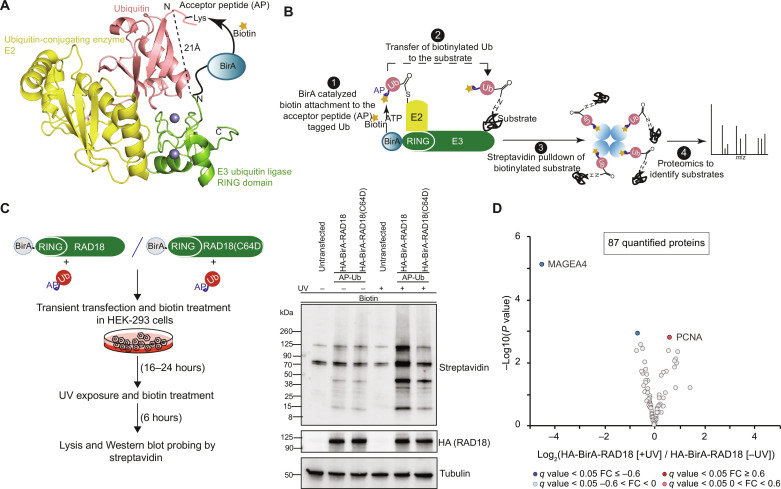
Using Ub-POD to identify substrates of RING E3 ligase RAD18. (**A**) Structural model of Ub (salmon) in complex with a Ub-conjugating enzyme E2 (yellow) and a Ub ligase E3 (RING) (green). The distance between the N termini of E3 and Ub is mentioned. Placing AP-tag at the N terminus of Ub and BirA at the catalytic end of RING-E3 ligase brings AP and BirA in proximity to each other. (**B**) Schematic representation of the Ub-POD method. (**C**) RAD18 Ub-POD workflow (left). HEK-293 cells transfected for 16 to 24 hours with AP-Ub and HA-BirA-RAD18 WT or HA-BirA-RAD18 C64D were exposed to UV (10 mJ/cm^2^) (right). Biotin was added at the time of transfection, and cells were allowed to recover 6 hours after UV irradiation. Untreated cells served as control. Lysates were subjected to SDS-PAGE and immunoblotting. Results are representative of two independent biological replicates. (**D**) Volcano plot of streptavidin pulldown enriched proteins isolated from cells transfected with HA-BirA-RAD18 WT and AP-Ub in the absence or presence of UV exposure (*n* = 3 biological replicates). Significantly altered proteins are shown in dark red or blue [false discovery rate (FDR) <0.05, log_2_FC >I0.6I] and light red or blue (FDR <0.05, 0 > log_2_FC < I0.6I) (moderated *t* test).

During Ub transfer to the target proteins, RING E3 ligases bind both substrate and E2~Ub thioester conjugate and mediate a direct transfer of Ub from the E2 to the substrate (*21*, *22*). Crystal structures of E2~Ub–RING E3 complexes have shown that RING E3s first activate the E2 ~ Ub thioester complex by forming a transient-intermediate complex between E2~Ub and the RING E3 and subsequently orient the E2~Ub thioester complex with respect to the substrate (*19*). In the UbcH5A~Ub-RNF4 complex structure reported by Plechanova *et al.* (*23*), we noted that the N terminus of the catalytic RING domain and N terminus of Ub point toward the same orientation into solvent and come in close proximity to each other (~21 Å) (fig. S1A). Similarly, analysis of the HECT NEDD4L~Ub structures (*24*, *25*) has revealed that the C terminus of the HECT ligase, which harbors the catalytic HECT domain, and the N terminus of Ub orient toward each other into the solvent (fig. S1B). These observations prompted us to develop a precise Ub-specific proximity-based labeling approach to identify the substrates of an E3 Ub ligase. Toward this, we tagged the catalytic end of an E3 Ub ligase using the WT *E. coli* biotin ligase enzyme (BirA) and the N terminus of Ub with BirA’s “acceptor peptide” (AP) (*26*) substrate (Fig. 1A). The proximity and the orientation alignment between the E3 catalytic domain and the N terminus of Ub in the catalytic intermediate complex will enable BirA to catalyze site-specific biotinylation of AP linked to Ub. Subsequently, the biotinylated AP-Ub is transferred onto the target proteins, allowing them to be efficiently isolated using streptavidin pulldown and identified through mass spectrometry (MS) (Fig. 1, A and B).

### Using Ub-POD to identify substrates of the RING E3 ligase RAD18

We first tested Ub-POD for substrate identification of the RING E3 ligase RAD18. Upon UV-induced bulky DNA lesions, RAD18, in a complex with the Ub-conjugating enzyme RAD6, mediates monoubiquitination of proliferating cell nuclear antigen (PCNA), thereby triggering translesion synthesis of DNA (*27*). Because RAD18 contains the catalytic RING domain at the N terminus, we tagged the N terminus of RAD18 with BirA to generate HA-BirA-RAD18, which we transiently expressed along with AP-Ub in human embryonic kidney (HEK) 293 cells in the presence of biotin. After 24 hours, cells were either exposed to UV or left untreated, followed by continued biotin treatment for 6 hours (Fig. 1C, left, described in detail in Materials and Methods). According to a previous study, maximal ubiquitination of PCNA was only observed 6 to 8 hours after irradiation (*28*). Cells were collected and lysed, and whole-cell lysates were subjected to SDS–polyacrylamide gel electrophoresis (SDS-PAGE) followed by immunoblotting with antistreptavidin antibody. Increased biotinylation clearly occurred in cells treated with UV relative to nontreated cells (Fig. 1C, right), indicating that Ub-POD can indeed monitor the activity of RAD18. Introduction of a point mutation (RAD18 C64D) that disrupts the interface of the RAD18-RAD6 complex resulted in substantial reduction in biotinylation (Fig. 1C, right). Moreover, expression of BirA alone or BirA-tagged RAD18 without AP-Ub only resulted in negligible biotinylation as compared to coexpression of these proteins together with AP-Ub (fig. S1C). Likewise, coexpression of AP-Ub with BirA alone only led to minimal biotinylation (fig. S1C). Together, these results indicated that BirA on its own does not notably biotinylate AP-Ub compared to catalysis-induced proximity between BirA-RAD18 and AP-Ub. Prompted by the positive outcome, we next used proteomics to identify these biotinylated proteins, which are possible ubiquitination substrates of RAD18. We followed similar experimental conditions as above, and the biotinylated proteins were isolated using streptavidin beads under denaturing conditions from both UV-treated and untreated cells in triplicates (see Materials and Methods). Quantitative MS analysis of the pulldown fractions was performed using TMTplex labeling. We identified PCNA as the most enriched candidate (Fig. 1D and table S1) in the UV-treated condition, providing proof of principle that the Ub-POD method can be used to identify substrates of a RING Ub ligase. Intriguingly, melanoma-associated antigen 4 (MAGEA4) was found enriched in the untreated condition (Fig. 1D and table S1). MAGEA4 has been recently identified as the stabilizer of RAD18 and was also shown to be ubiquitinated by RAD18 in vitro (*29*). Our data indicate that RAD18 likely ubiquitinates MAGEA4 when cells are not exposed to UV but preferentially ubiquitinates PCNA after UV exposure. In the next step, we sought to improve the specificity and sensitivity of substrate identification using Ub-POD to increase the number of identified proteins and to boost the enrichment of substrates.

### A variant of the AP tag improves the sensitivity of Ub-POD

**Fig. 2.**
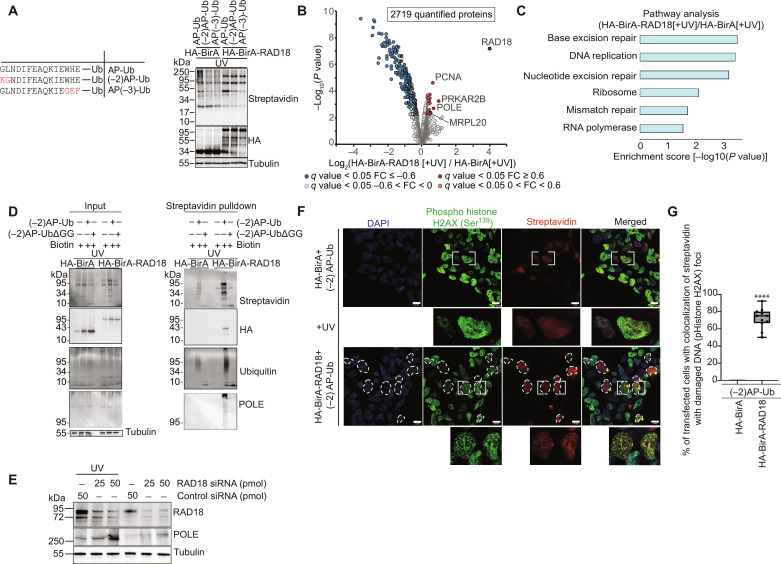
A variant of AP-tag increases the efficiency of Ub-POD. (**A**) Left: Sequences of various AP-tag variants. Right: AP-tag variants were expressed in HEK-293 cells along with HA-BirA or HA-BirA-RAD18. Biotin was added at the time of transfection, and cells were allowed to recover 6 hours after UV irradiation. Lysates were subjected to SDS-PAGE and immunoblotting. Results are representative of three independent biological replicates. (**B**) HEK-293 cells were transfected with (−2)AP-Ub and HA-BirA or HA-BirA-RAD18 followed by UV exposure (10 mJ/cm^2^). Cells were treated the same as in (A). Volcano plot of streptavidin enriched proteins in the two abovementioned conditions (*n* = 3 biological replicates). Significantly altered proteins are shown in dark red or blue (FDR <0.05, log_2_FC >I0.6I) and light red or blue (FDR <0.05, 0 > log_2_FC < I0.6I) (moderated *t* test). (**C**) GO term analysis of potential RAD18 ubiquitination substrates. Bar graph shows significantly enriched pathways. (**D**) HEK-293 are transfected with indicated plasmids. Cells were treated, and lysates were immunoblotted the same as in (A). Results are representative of two independent biological replicates. (**E**) HEK-293 cells were transfected with indicated small interfering RNAs (siRNAs). After 48 hours, cells were exposed to UV (10 mJ/cm^2^). The UV-treated and untreated cells were allowed to recover for 6 hours. Lysates were subjected to SDS-PAGE followed by immunoblotting with the indicated antibodies. Results are representative of three independent biological replicates. (**F**) Confocal microscopy of HEK-293 cells expressing either HA-BirA or HA-BirA-RAD18, along with (−2)AP-Ub, immunostained with anti–phospho histone H2AX (Ser^139^) (green), antistreptavidin (red). Cells with notable Streptavidin signal are circled using white dotted lines. Scale bar, 12 μm. DAPI, 4′,6-diamidino-2-phenylindole. (**G**) Percentage of transfected cells showing colocalization (Pearson’s coefficient > 0.5) of streptavidin and phospho histone H2AX (Ser^130^) signals (indicated by yellow arrows) is represented (****P < 0.0001) (*n*, biological replicates = 2, 10 different fields).

Because of a relatively high intrinsic affinity of AP for BirA, protein-protein interaction–independent biotinylation may occur in cells, giving rise to background biotinylation of free AP-Ub that is not primed for ubiquitination by the BirA-tagged Ub ligase. Fernández-Suárez *et al.* (*30*) tested multiple variants of the AP sequences for their ability to specifically get biotinylated strictly dependent on the proximity to the BirA-labeled protein. Some of these variants included the deletion of up to three amino acids from one or both ends of AP to decrease its interaction surface area with BirA. On this basis, we prepared two variants of AP-Ub: (−2)AP-Ub in which two N-terminal residues from AP were removed and AP(−3)-Ub in which C-terminal 3 residues of AP were deleted (Fig. 2A). WT as well as truncated AP-Ub constructs were overexpressed in HEK-293 cells, along with BirA or BirA-RAD18, followed by biotin treatment and UV exposure (Fig. 2A). In agreement with previous reports, both truncated AP variants, (−2)AP-Ub and AP(−3)-Ub, showed much lower background biotinylation, in the presence of BirA, compared to AP-Ub. While AP-Ub as well as both variants showed increased biotinylation in the presence of BirA-RAD18 compared to BirA, the relative increase in biotinylation between BirA-RAD18 and BirA alone was more prominent in the presence of (−2)AP-Ub or AP(−3)-Ub than with AP-Ub (Fig. 2A). This indicated that both (−2)AP-Ub and AP(−3)-Ub are more suitable for Ub-POD compared to AP-Ub. Fernández-Suárez *et al.* (*30*) showed that the proximity-dependent biotinylation of AP(−3) is less pronounced because of increased Michaelis constant (*K*_M_) (350 μM) of AP(−3) compared to AP (*K*_M_ = 25 μM), indicating that AP(−3) binds much weaker to BirA. AP(−3) also exhibited a diminished Turnover number (*k*_cat_) (0.5 min^−1^) compared to AP (14 min^−1^), and it was proposed that AP(−3) tag is only suitable to detect protein-protein interactions with a half-life of more than a minute. Because ubiquitination reactions are typically much faster than the kinetics of AP(−3) biotinylation by BirA (*26*, *30*–*32*), we chose (−2)AP-Ub for our subsequent Ub-POD experiments.

When (−2)AP-Ub was used in Ub-POD of RAD18, the overall number of identified hits increased in the MS analysis compared to the experiment performed with AP-Ub ( Figs. 1D and 2B and table S2). BirA and (−2)AP-Ub coexpression was used as a control in this experiment as it showed negligible background biotinylation (Fig. 2A). Gene ontology (GO) enrichment analysis of RAD18 Ub-POD hits highlighted GO terms related to base excision repair (*P* ≤ 0.001), nucleotide excision repair (*P* ≤ 0.001), mismatch repair (*P* ≤ 0.01), and DNA replication (*P* < 0.001), which are highly relevant to the role of RAD18 in UV-induced DNA damage repair (Fig. 2C). Because RAD18 C64D showed considerably lower background of biotinylation compared to RAD18 WT (Fig. 1C), we next probed whether RAD18 C64D can serve as a control in RAD18 Ub-POD experiments. Unexpectedly, only one protein was identified as a significant hit [false discovery rate (FDR) < 0.05, LogFC >0.6] in RAD18 WT compared to RAD18 C64D (fig. S2A). The enrichment of [log_2_ fold change (FC)] PCNA in RAD18 C64D was only slightly lower compared to RAD18 WT. As a result, PCNA was not identified as a significant hit in the comparison between RAD18 WT and RAD18 C64D (fig. S2, A and B, and table S2) but unexpectedly, PCNA was found enriched in Ub-POD experiment performed with RAD18 C64D (fig. S2B). Because RAD18 is known to dimerize (*33*), exogenously expressed HA-BirA-RAD18 C64D might dimerize with endogenous RAD18, making this mutant a suboptimal control for proximity-dependent proteomics experiments. Because many RING E3 ligases are known to dimerize (*34*), the use of activity-deficient point mutants as controls in Ub-POD experiments will need to be carefully considered and compared to BirA alone.

Among the identified RAD18 Ub-POD hits (Fig. 2B), DNA polymerase epsilon (Pol ε) catalytic subunit POLE was of particular interest to us, as this is a predicted substrate of RAD18 with a high confidence score in Ubibrowser (*5*, *35*). Pol ε is crucial for optimal leading-strand DNA synthesis, and Pol ε–mediated leading-strand replication is dependent on the sliding-clamp processivity factor PCNA (*36*). In the presence of DNA lesions, monoubiquitination of PCNA by RAD18 upon replication stress results in switching of replicative polymerases (Pol ε and Pol δ) with translesion synthesis polymerases. A previous diGly proteomics analysis aiming to identify DNA damage–induced ubiquitination events revealed that ubiquitination of POLE is up-regulated upon UV exposure (*37*). To validate that POLE is indeed ubiquitinated by RAD18 upon UV exposure, we overexpressed either BirA or BirA-RAD18 in HEK-293 cells along with (−2)AP-Ub and treated these cells with UV. As a control experiment, we also coexpressed BirA or BirA-RAD18 with unconjugatable (−2)AP-UbΔGG lacking the two terminal glycines. Immunoblotting of the streptavidin-pulldown fractions with antibodies recognizing POLE showed the appearance of a high molecular POLE species only when BirA-RAD18 and (−2)AP-Ub are coexpressed (Fig. 2D). This experiment also shows that Ub-POD is highly Ub specific as coexpression of BirA-RAD18 with (−2)AP-UbΔGG fails to show any notable biotin or Ub smear signal in the Western blotting experiments (Fig. 2D). To further test whether the modification of POLE is indeed ubiquitination, we performed another Ub-POD experiment with BirA-RAD18 in the presence of UV. Here, we isolated the streptavidin-bound fraction from lysates and treated it with the deubiquitinase (DUB) USP2, which removes Ub molecules attached to a substrate (fig. S2C). Streptavidin-bound fraction showed high molecular POLE species that disappears upon USP2 treatment. This indicates that POLE is ubiquitinated under these conditions. We next used small interfering RNA (siRNA) to silence the expression of RAD18 and tested the effect of this on the stability of POLE (Fig. 2E). Silencing of RAD18 increased cellular POLE levels in a largely UV-dependent manner. Hence, apart from monoubiquitinating PCNA upon UV-induced DNA damage, RAD18 seems to be involved in polyubiquitination of POLE. Future studies are required to understand the exact nature and significance of this intriguing new ubiquitination event in the context of the various DNA repair pathways that RAD18 plays a role in (*28*, *38*–*40*).

### The effect of Ub recycling on Ub-POD

The process of ubiquitination is reversible through the action of the DUB family of enzymes (*41*, *42*). Cellular activity of DUB enzymes could potentially lead to the recycling of biotinylated (−2)AP-Ub used in the Ub-POD experiment, compromising the specificity and efficiency of substrate identification. To understand the impact of Ub recycling on the Ub-POD method, we used a nonselective, broad spectrum DUB inhibitor PR619 [2,6-diaminopyradine-3,5-bis(thiocynate)] that efficiently silences four of the five DUB families (*43*). We treated BirA-RAD18–transfected cells with 10 μM PR619, 2 hours after UV exposure. The control BirA-transfected cells were not treated with PR619. Proteomics analysis showed that PR619 treatment increases the efficiency of PCNA identification when compared to the earlier experiment (Fig. 2B) performed without PR619 treatment (fig. S3, A and B, and table S3). PR619 treatment also increased the overall number of proteins identified as hits, and GO annotation analysis showed that there is enrichment of proteins involved in the process of DNA replication (*P* ≤ 0.01) (fig. S3, B and C). We identified several proteins that are predicted to be RAD18 substrates by the repositories such as Ubibrowser (*5*), BioGRID (*44*), and Reactome (*45*), and these candidates have been highlighted in table S3. Our data indicate that a pan DUB inhibitor treatment could improve substrate identification by Ub-POD as executed here for RAD18.

### Exploiting Ub-POD to monitor spatial ubiquitination

Visualizing where in the cell ubiquitination of substrates is taking place adds cellular context to understanding the function of a given ligase. Ub-POD experiments coupled to streptavidin immunofluorescence and confocal microscopy can, in principle, reveal the cellular localization of ubiquitinated substrates. Taking advantage of the fact that RAD18-mediated PCNA monoubiquitination and the following polymerase switching event take place at UV irradiation–induced RAD18 nuclear foci (*28*), we addressed whether Ub-POD can visualize RAD18-mediated ubiquitination at the damaged DNA foci. In irradiated cells expressing BirA-RAD18 and (−2)AP-Ub, biotin colocalized with phosphorylated Histone H2AX (Ser^139^) (γ-H2AX), which is a bona fide marker of ionizing radiation–induced DNA damage (*46*) (Fig. 2, F and G). However, we cannot differentiate the biotin signal coming from the autoubiquitinated RAD18 and ubiquitinated substrates such as PCNA or POLE at this point. Further experiments with a RAD18 variant defective in autoubiquitination are needed to decouple autoubiquitination and substrate ubiquitination events. As expected, biotin and HA (which detect HA-BirA or HA-BirA-RAD18) colocalized in nuclear puncta only in HA-BirA-RAD18–expressing cells (fig. S4). Together, these results indicated that Ub-POD can indeed provide insights into the cellular localization of a given E3 ligase–mediated ubiquitination.

### Using Ub-POD on the RING E3 ligase TRAF6

**Fig. 3.**
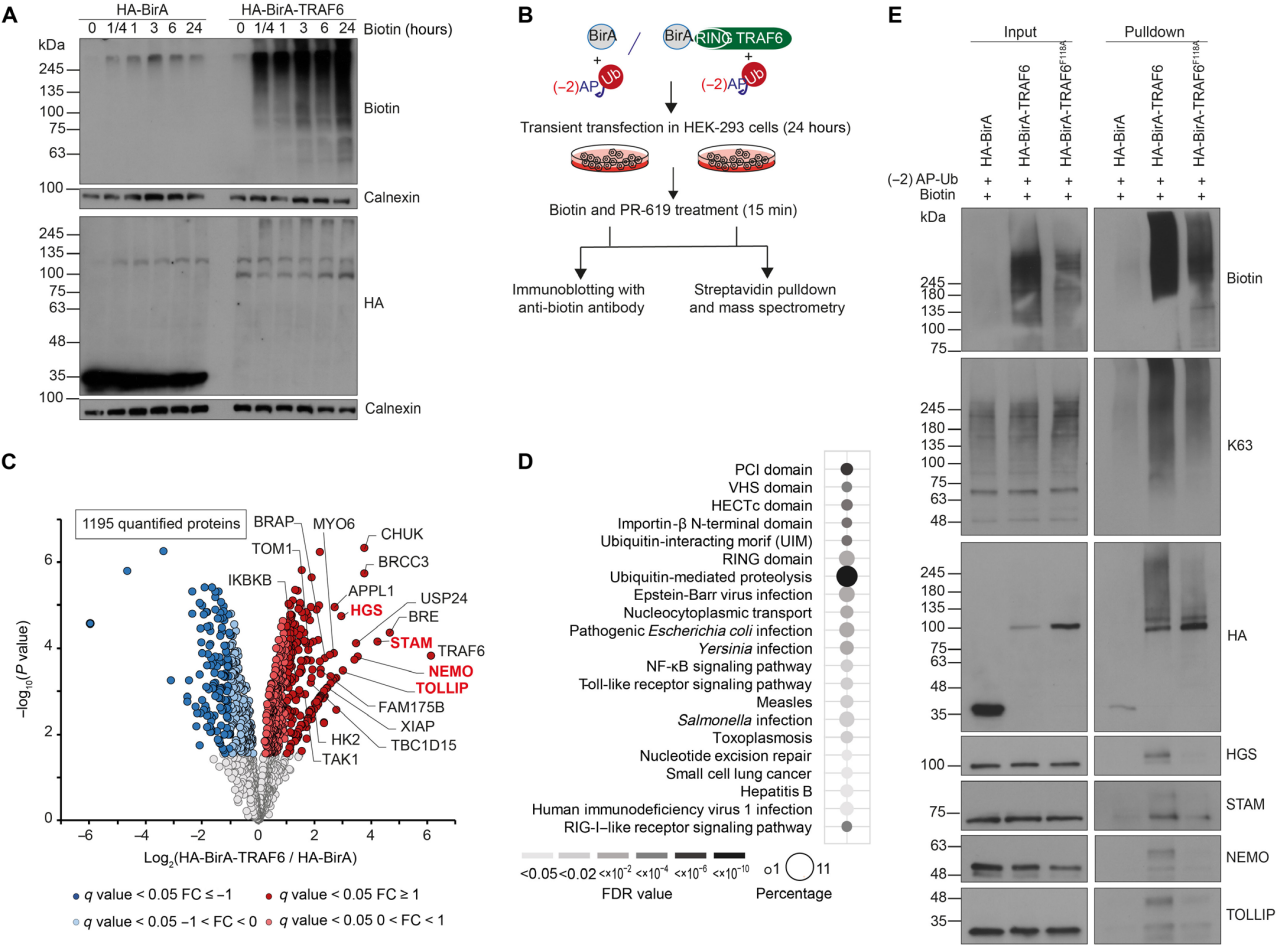
Using Ub-POD on the RING E3 ligase TRAF6. (**A**) Biotin time course to estimate appropriate biotin treatment duration. HEK-293 cells were transfected with (−2)AP-Ub together with HA-BirA or HA-BirA-TRAF6 for 24 hours. Biotin (100 μM) was either added together with transfection mixture for 24 hours or added 6 hours, 3 hours, 1 hour, or 15 min before harvest, respectively. Notably, 15-min treatment was sufficient to induce strong biotinylation in HA-BirA-TRAF6 overexpressing cells and was therefore kept as treatment condition throughout all experiments. (**B**) Experimental setup for Ub-POD-TRAF6-MS. HEK-293 cells transiently overexpressing (−2)AP-Ub together with HA-BirA or HA-BirA-TRAF6 for 24 hours were subjected to biotin (100 μM) and PR-619 (10 μM) for 15 min and harvested. An aliquot was reserved for analysis via immunoblotting, the residual lysate was prepared for streptavidin pulldown and subjected to MS (*n* = 4 biological replicates). (**C**) Volcano plot depicting altered biotinylated proteins after enrichment via streptavidin pulldown from HA-BirA-TRAF6 or HA-BirA expressing HEK-293 cells. Known and selected potential TRAF6 substrates are labeled. Validated hits depicted in 3E are highlighted in red. Significantly altered proteins are shown in dark red or blue (FDR < 0.05, log_2_FC > I1I) and light red or blue (FDR < 0.05, 0 > Ilog_2_FCI < I1I) (moderated *t* test, *n* = 4 independent experiments); (**D**) GO terms of proteins found to be significantly enriched in HA-BirA-TRAF6 versus HA-BirA with FC ≥1. Dot size correlates to number of proteins, dot color to term enrichment (FDR value). (**E**) Validation of TRAF6 substrates identified by Ub-POD-TRAF6-MS. HEK-293 cells transiently overexpressing (−2)AP-Ub together with HA-BirA, HA-BirA-TRAF6, or dimerization mutant HA-BirA-TRAF6F118A for 24 hours were subjected to biotin (100 μM) and PR-619 (10 μM) for 15 min. Lysates were subjected to streptavidin pulldown followed by SDS-PAGE and immunoblotting.

We next sought to benchmark our approach with the well-studied RING E3 ligase TRAF6, which mediates K63 Ub linkage types in concert with the Ub-conjugating enzyme heterodimer UBE2N-UBE2V1. TRAF6 bridges TNF receptor activation via exogenous agents or endogenous mediators and subsequent activation of transcriptional responses via NF-κB and mitogen-activated protein kinase pathways (*47*, *48*). First, we performed a biotinylation time course to shorten biotin incubation and reduce nonspecific labeling. We transiently cotransfected HEK-293 cells with (−2)AP-Ub and BirA-TRAF6 or BirA alone for 24 hours and grew cells for up to 24 hours in the presence of biotin. While BirA was expressed at substantially higher levels than BirA-TRAF6, cells expressing the former did not show any biotinylation irrespectively of the biotin incubation time. In contrast, biotinylation was clearly detectable in BirA-TRAF6–expressing cells 15 min after biotin addition (Fig. 3A). Because signaling downstream of TRAF6 can be observed between 5 and 20 min after pathway activation (*49*), we settled on 15 min for all future labeling experiments to capture ubiquitination substrates.

To further examine the specificity of the Ub-POD method, we compared biotinylation levels of TRAF6 WT, the dimerization mutant TRAF6 F118A, and the catalytically dead mutant TRAF6 C70A (*50*, *51*). As expected, both mutants showed a substantial decrease in biotinylation despite their higher expression compared to TRAF6 WT (fig. S5A). We speculate that residual biotinylation might stem from endogenous TRAF6, which dimerizes with overexpressed TRAF6 mutants.

To identify potential TRAF6 substrates, we transiently cotransfected HEK-293 cells with (−2)AP-Ub and N-terminally tagged BirA-TRAF6 or BirA alone for 24 hours and subsequently treated cells with biotin and PR-619 for 15 min (Fig. 3B). Following lysis, we removed an aliquot from each replicate to confirm induction of biotinylation (fig. S5B). Notably, overexpression of TRAF6 was sufficient to induce downstream NF-κB signaling, possibly because of enhanced dimerization of TRAF6 that is crucial for its autoubiquitination and catalytic activity (*50*). We subjected these samples to streptavidin pulldown under denaturing condition, on-beads tryptic digestion, and MS analysis. Across the different conditions, quadruplicate samples showed high Pearson correlation (*r* ≥ 0.928). In total, we identified 1944 proteins of which 1195 proteins passed stringent filtering (quantification with at least two unique peptides in three of four replicates). A total of 395 proteins were significantly enriched in BirA-TRAF6–expressing cells compared to HA-BirA control–expressing cells; 182 of these proteins reached the threshold of FDR <0.05 and FC >1 (Fig. 3C and tables S4 and S5). GO annotation analysis of these candidates unveiled shared protein motifs, such as E3 ligase domains (RING and HECT) and Ub-interacting motifs (UIM). Intriguingly, specific enrichment of proteins involved in immune response pathways against bacterial and viral infection, including Toll-like receptor and NF-κB signaling pathway, among others, gives us confidence in the robustness of our approach even in nonimmune cell types such as HEK-293 cells (*48*, *51*) (Fig. 3D). TRAF6 is the strongest regulated hit possibly because of its autoubiquitination in the active, oligomerized state (*50*). Furthermore, our approach enabled the specific enrichment of bona fide TRAF6 substrates [NF-kappa-B essential modulator (NEMO) and TGF-beta-activated kinase 1 (TAK1)] (*51*, *52*). We also enriched for the deubiquitinating enzyme complex [BRCC36 isopeptidase complex (BRISC)], which is specific for Lys^63^-linked Ub hydrolysis (*53*). Furthermore, strongly regulated hits are VPS-27, Hrs and STAM (VHS) -domain containing and Ub-binding proteins located at endosomes [Signal transducing adapter molecule 1 (STAM), Hepatocyte growth factor-regulated tyrosine kinase substrate (HGS), and Target of Myb1 membrane trafficking protein (TOM1)], which are involved in receptor tyrosine kinase–mediated endocytosis (*54*). Next, we sought to validate potential new TRAF6 substrates. Candidates were labeled in cells by BirA-TRAF6 and enriched via streptavidin pulldown before their detection by immunoblotting. Notably, BirA-TRAF6 F118A– and BirA-only–expressing cells served as negative controls. Using this approach, we were able to confirm the known TRAF6 substrate NEMO whose biotinylation was absent in BirA-only– and in BirA-TRAF6 F118A–expressing cells despite substantial higher expression levels of these control proteins (Fig. 3E). We also confirmed TRAF6 labeling–dependent enrichment of the endosomal sorting complexes required for transport (ESCRT) protein STAM and HGS as well as of the selective autophagy receptor Toll-interacting protein (TOLLIP), all of which were identified as potential new substrates of TRAF6 (Fig. 3E). In addition, we observed strong enrichment of K63-linked polyubiquitylated proteins, suggesting that substrate candidates might be modified by this TRAF6 signature Ub chain type. Similar as described for RAD18, we confirmed ubiquitination of NEMO, STAM, HGS, and TOLLIP by two ortholog experimental strategies using (−2)AP-UbΔGG as a negative control for in cell biotinylation reactions (fig. S5C) and USP2 treatment for streptavidin pulldowns (fig. S5D). Together, these results show that our Ub-POD approach can identify substrate candidates of different RING ligases in distinct stress conditions.

### Using Ub-POD to identify substrates of the U-box–containing ligase CHIP

**Fig. 4.**
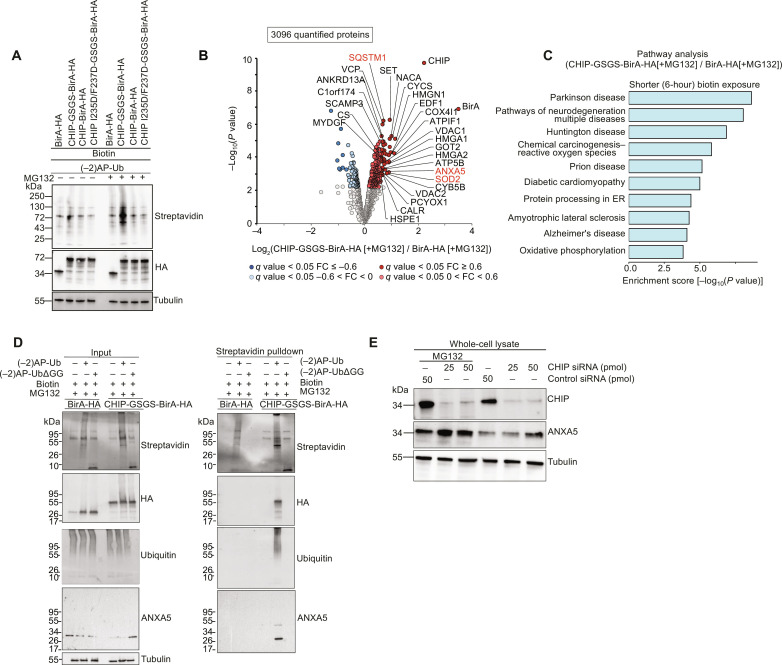
Using Ub-POD to identify substrates of U-box ligase CHIP. (**A**) HEK-293 cells transfected for 16 to 24 hours with (−2)AP-Ub and indicated BirA-tagged constructs. Cells were treated with MG132 (10 μM) for 6 hours. Cells were kept in biotin (100 μM) the whole time. Lysates were separated by SDS-PAGE and analyzed by Western blotting. Results are representative of two independent biological replicates. (**B**) HEK-293 cells transfected for 24 hours with (−2)AP-Ub and BirA-HA or CHIP-GSGS-BirA-HA were incubated with biotin (100 μM) and MG132 (10 μM) for 6 hours. Lysates were subjected to streptavidin pulldown and MS analysis. Volcano plot of proteins labeled by CHIP-GSGS-BirA-HA and HA-BirA in 6 hours (*n* = 3 biological replicates). Significantly altered proteins are shown in dark red or blue (FDR <0.05, log_2_FC > I0.6I) and light red or blue (FDR <0.05, 0 > log_2_FC < I0.6I) (moderated *t* test). (**C**) Bar graph representation of enriched GO terms for candidate CHIP substrates. (**D**) HA-BirA or CHIP-GSGS-BirA-HA transfected HEK-293 cells were cotransfected with either (−2)APUb or (−2)AP-UbΔGG. After 6 hours of MG132 treatment, whole-cell lysates were prepared followed by streptavidin pulldown. Pulldown and inputs were run on SDS-PAGE followed by immunoblotting with indicated antibodies. Results are representative of two independent biological replicates. (**E**) Knockdown of CHIP and immunoblotting for CHIP and ANXA5: HEK-293 cells were reverse transfected with either control siRNA (50 pmol) or different concentrations of CHIP siRNA (25 or 50 pmol). After 48 hours, cells were treated with either vehicle (dimethyl sulfoxide) or MG132 (10 μM). Whole-cell lysates, prepared after 6 hours of treatment, were run on SDS-PAGE followed by immunoblotting with the indicated antibodies. Results are representative of three independent biological replicates.

Next, we asked whether Ub-POD can be applied to a U-box domain–containing Ub ligase. The U-box domain adopts the same fold as the RING domain but lacks the Zn^2+^ coordination residues, which are replaced by a tight hydrogen bonding network, contributing to the integrity of the catalytic domain structure (*55*, *56*). CHIP, which is also called STUB1, is a U-box domain–containing Ub ligase that functions as a key regulator of the protein quality control machinery. CHIP binds the molecular chaperones Hsc70, Hsp70, and Hsp90 through its N-terminal tetratricopeptide repeat domain and facilitates the polyubiquitination of misfolded client proteins via its C-terminal catalytic U-box (*57*–*59*). We used Ub-POD on CHIP to benchmark our approach for a U-box E3 ligase and to possibly identify new substrates of CHIP. In line with the concept of Ub-POD, we prepared a CHIP construct where BirA is tagged to the C terminus of CHIP (CHIP-BirA), because the catalytic U-box domain of CHIP is located at the extreme C terminus of the protein (Fig. 4A). We also designed another construct in which we inserted a GSGS linker between the catalytic U-box domain of CHIP and BirA (CHIP-GSGS-BirA) (Fig. 4A), under the assumption that providing more degrees of freedom for BirA may improve BirA-mediated site-specific biotinylation of (−2)AP-Ub leading to more efficient target protein biotinylation. CHIP-BirA or CHIP-GSGS-BirA were transiently expressed in HEK-293 cells for 16 to 24 hours along with (−2)AP-Ub. As CHIP directs target proteins for proteasomal degradation, we treated cells with the proteasome inhibitor MG132 to preserve substrates for the duration of the experiment. Untreated cells served as control. Streptavidin immunoblotting of whole-cell lysates showed that in the presence of MG132, expression of both CHIP-BirA and CHIP-GSGS-BirA resulted in a notable increase in biotinylation (Fig. 4A). CHIP-GSGS-BirA showed more biotinylation compared to CHIP-BirA, suggesting that the linker improves the efficiency of substrate biotinylation, at least in the case of CHIP (Fig. 4A). We have not tried to vary the linker length here, and we expect that the linker type and length connecting the BirA and the ligase might need to be optimized for each ligase although a flexible linker such as GSGS should provide a good starting point. Because the structure of CHIP in complex with E2 is known (*60*), we designed a mutant of CHIP (I235D/F237D) that is deficient in binding to E2 as a negative control (Fig. 4A). As expected, the biotinylation is notably lower in the condition where CHIP I235D/F237D-GSGS-BirA is transfected compared to CHIP WT-GSGS-BirA.

We chose CHIP-GSGS-BirA for the identification of potential substrates through proteomics. We performed the Ub-POD transfections as above and used expression of BirA alone as a control. Notably, cells in both conditions were treated with MG132. We did not induce proteotoxicity in this experiment, because we sought to identify substrates that get ubiquitinated by CHIP under basal conditions. In this particular experiment, no other identified protein except CHIP met our stringent FDR < 0.05 cutoff (fig. S6A and table S6), but our analysis showed that these data still contained relevant information when only *P* value <0.05, logFC >0.75 were used as a criterion to highlight the enriched proteins. Hypoxia-inducible transcription factor, HIF1A, a well-known substrate of CHIP (*61*), was specifically identified in CHIP-GSGS-BirA–expressing cells (fig. S6A and table S6). In addition, CHIP is reported to tag proteasomal subunits with polyubiquitin chains and target them to aggresomes (*62*). In agreement with this, several proteasomal subunit proteins were also identified as hits in the MS analysis (fig. S6A and table S6). Several neurodegenerative diseases, e.g., Parkinson’s disease, spinocerebellar ataxia, Alzheimer’s disease, and Huntington’s disease (*P* < 0.0001), were found enriched in GO term analysis in agreement with the known physiological role of CHIP (fig. S6B).

Because CHIP is involved in several different cellular processes apart from protein quality control (*63*), we wondered whether we could identify an expanded or a different set of CHIP substrates by reducing the exposure of biotin in CHIP-GSGS-BirA–expressing cells. Therefore, instead of exposing the cells to biotin immediately after transfection for 24 hours, here, we added biotin to cells 24 hours after transfection with CHIP-GSGS-BirA and (−2)AP-Ub for 6 hours along with MG132 (Fig. 4B). Two known substrates of CHIP, Sequestosome-1 (SQSTM1) (also known as p62) (*64*) and superoxide dismutase 2 (SOD2) (*65*), were identified (Fig. 4B and table S7) with this shorter biotin exposure. Notably, changes in the substrate identification profile depending on the duration of biotin exposure could be due to the different half-lives of various substrates of CHIP. GO annotation analysis of hits highlighted processes relevant to known functions of CHIP, such as pathways of neurodegeneration (*P* ≤ 0.0001), and protein processing in the endoplasmic reticulum (*P* ≤ 0.01) (Fig. 4C).

Because we have used MG132 in both CHIP-GSGS-BirA as well as BirA expression conditions so far, the identified substrates may not necessarily be the ones that are strictly regulated through the proteasome (fig. S6A and Fig. 4B). Therefore, we next performed a comparison of Ub-POD CHIP in the presence or absence of MG132 (fig. S6, C and D, and table S8) to identify CHIP substrates targeted for proteasomal degradation. Previously known substrates of CHIP such as SQSTM1 (*64*), NPM1 (*66*), PSMD4 (*67*), and HDAC6 (*68*) were identified in this Ub-POD analysis (table S8). GO annotation analysis showed Ub-mediated proteolysis as the most significantly enriched pathway (*P* < 0.0001) in the presence of MG132 (fig. S6, C and D). Applying Ub-POD to CHIP demonstrated that a flexible linker between BirA and the candidate Ub ligase can improve the identification of substrates and that biotin exposure times and stimuli may need to be optimized for a given Ub ligase to identify different sets of substrates.

Comparison of the putative CHIP substrates identified by Ub-POD to the ones previously identified through an OUT screen (*15*) revealed that, among the 308 potential CHIP substrates identified by Ub-POD, 25 proteins overlapped with the 226 putative CHIP substrates found in the OUT screen (fig. S6F and table S9). Of these 25 overlapping proteins, only SQSTM1 and CTNNB1 were already known substrates of CHIP. However, two other well-known substrates of CHIP, SOD2 and HIF1, which were identified by Ub-POD, were not found in the OUT screen for CHIP.

Moreover, annexin 5 (ANXA5), a member of the annexin superfamily of proteins, was identified by Ub-POD as well as by OUT screen (Fig. 4B). Annexins are a family of cytosolic proteins that translocate to the plasma membrane in a calcium-dependent manner and thus regulates actin cytoskeleton dynamics (*69*). Intriguingly, a previous report found that ANXA5 is up-regulated in CHIP knockout cortical neurons compared to CHIP-expressing neurons (*70*). On this basis, we hypothesized that ANXA5 is a possible substrate of CHIP ligase. To test this, we overexpressed either BirA or CHIP-GSGS-BirA in HEK-293 cells along with (−2)AP-Ub or (−2)AP-UbΔGG (Fig. 4D). Biotinylated proteins were enriched using streptavidin pulldown and probed by anti-ANXA5 antibody (Fig. 4D). We observed specific enrichment of ANXA5 only in conditions where CHIP-GSGS-BirA and (−2)AP-Ub were coexpressed. ANXA5 was not enriched when (−2)AP-UbΔGG was expressed in our experiment pointing again to the highly Ub-specific nature of our proximity labeling strategy. In our streptavidin pulldown, we noticed that unmodified form of ANXA5 was also pulled down in substantial amounts. This could either be due to the direct biotinylation of ANXA5 in the Ub-POD experiment independent of Ub attachment or because of the previously reported polymeric nature of ANXA5 that might have resulted in the coelution of modified and unmodified forms of ANXA5 in our experiment (*71*). To dissect this, we treated the streptavidin pulldown fractions of our CHIP Ub-POD experiment with USP2 (fig. S6G). USP2 treatment led to disappearance of both high–molecular weight bands of ANXA5 and the unmodified form of ANXA5, effectively ruling out the possibility that ANXA5 was biotinylated in a Ub-independent manner. Toward further validation of ANXA5 regulation through CHIP, we used siRNA to knockdown CHIP in ANXA5 in HEK cells (Fig. 4E). Knockdown of CHIP leads to accumulation of ANXA5 in a dose- and proteasomal-dependent manner. Our data from studying three representative E3 ligases (Figs. 1 to 4) indicate that Ub-POD is highly Ub specific and is capable of identifying novel substrates of Ub ligases.

### Comparison of BioID and Ub-POD for E3 ligase substrate identification

**Fig. 5.**
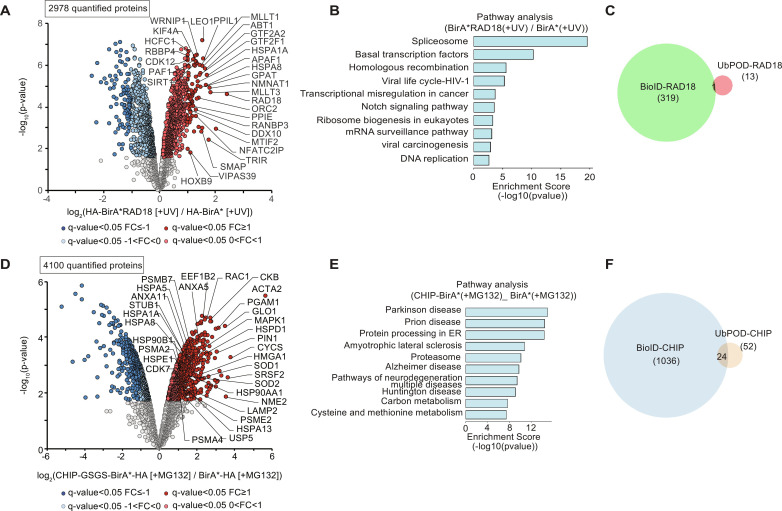
BioID of RAD18 and CHIP. (**A**) BioID analysis of RAD18. HEK-293 cells transfected for 24 hours with BirA* or BirA*-RAD18 were exposed to UV (10 mJ/cm^2^) and allowed to recover for 6 hours. Cells were kept in 100 μM biotin the whole time. Lysates were subjected to streptavidin pulldown followed by MS (*n* = 3 biological replicates). Volcano plot of proteins labeled by BirA*-RAD18 and BirA*. Significantly altered proteins are shown in dark red or blue (FDR <0.05, log_2_FC > I1I) and light red or blue (FDR <0.05, 0 > log_2_FC < I1I) (moderated *t* test). (**B**) Bar graph depicting significantly enriched GO terms of hits from (A). (**C**) Overlap between hits identified in RAD18 BioID and Ub-POD experiments. (**D**) BioID analysis of CHIP. HEK-293 cells transfected for 24 hours with BirA* or CHIP-GSGS-BirA* were incubated with MG132 (10 μM) for 6 hours. Cells were kept in 100 μM biotin the whole time. Streptavidin pulldowns were performed with lysates followed by MS analysis (*n* = 3 biological replicates). Volcano plot of proteins labeled by CHIP-GSGS-BirA* and BirA*. Significantly altered proteins are shown in dark red or blue (FDR <0.05, log_2_FC >I1I) and light red or blue (FDR <0.05, 0 > log_2_FC < I1I) (moderated *t* test). (**E**) Bar graph representation of significantly enriched GO terms with hits identified in (D). (**F**) Overlap between hits identified in CHIP BioID and Ub-POD experiments.

Proximity labeling approaches such as BioID, TurboID, and AirID have been used before to identify transient protein-protein interactions (*72*–*74*). These methods depend on a mutated form of BirA (BirA* which has R118G mutation) or other engineered biotin ligases fused to the protein of interest (POI) to release highly reactive adenosine monophosphate (AMP)–biotin into the surrounding solvent and label the neighboring macromolecules with biotin. Unlike BirA*, which is used in BioID, WT BirA used in Ub-POD does not ligate biotin nonspecifically to all the spatially proximal proteins but only attaches biotin to the (−2)AP-Ub that is in close proximity to E3. We compared the suitability of BioID and Ub-POD for the identification of E3 ligase substrates. Thereto, RAD18 was tagged at its N terminus with BirA* (*73*, *75*). BirA* alone or BirA*-tagged RAD18 were expressed in HEK-293 cells. UV exposure and biotin treatment in the BioID experiment were followed exactly as for Ub-POD. Proteomics analysis showed that BioID-RAD18 identified numerous neighboring proteins of RAD18 (Fig. 5A and table S10). Functional annotation analysis of BioID-RAD18 hits revealed significant enrichment of GO categories related to spliceosome, basal transcription factors, and homologous recombination (*P* < 0.0001) (Fig. 5B). Compared to the 13 substrate candidates identified in RAD18 Ub-POD, BioID-RAD18 identified 320 proximity partners (Fig. 5C), providing a complex snapshot of RAD18’s many neighboring and potential interacting proteins. As expected on the basis of the different labeling strategies, only one common protein (RAD18) was found to be significantly enriched when comparing BioID and Ub-POD experiments of RAD18 (Fig. 5, A to C). To further explore the differences in BioID and Ub-POD, we also performed BioID analysis for CHIP. CHIP was tagged with BirA* at its C-terminal end (CHIP-BirA*). MG132 and biotin treatment was kept the same for BioID-CHIP as for CHIP Ub-POD (Fig. 4B). GO pathway analysis of the hits identified by CHIP-BirA* compared to BirA* unveiled pathways related to various cellular roles of CHIP with significant *P* values (*P* < 0.0001) (Fig. 5, D and E). Among the proximity partners were heat shock proteins, proteasomal subunit proteins, and several known CHIP substrates (SOD2, SOD1, CDK4, and ANXA5) (Fig. 5D and table S11). Comparison of all 1060 proximity partners that were found enriched in BioID-CHIP (Fig. 5D) with the 52 substrate candidates identified by CHIP Ub-POD (Fig. 4B and fig. S6A) showed 24 common hits between these two datasets (Fig. 5F and table S12). These results indicated that, though both BioID and Ub-POD are dependent on proximity-based biotinylation of target proteins, Ub-POD identifies far fewer number of hits compared to BioID likely because it is specific to the Ub pathway proteins and substrates.

## DISCUSSION

Here, we developed a new approach to effectively label the substrates of a given Ub ligase with biotin directly in cells and identify them using quantitative MS. It is worth mentioning that our approach has important differences from the traditional proximity labeling approaches such as BioID and TurboID, which have been successfully used in a number of studies to identify interacting partners of a POI (*72*, *73*). The BioID method involves the expression of the POI tagged with a mutant of BirA, called BirA* or BioID, which bears the mutation R118G in the active site of the enzyme. BirA* releases highly reactive biotin-AMP conjugate into solvent that can label any protein within the spherical radius of ~10 nm around POI, thus providing a clear picture of the interactors and the environment of the POI. However, BioID does not differentiate between the direct interactor and any other protein within the vicinity of the POI. In the context of E3 ligases, BioID would also not be able to differentiate between the substrate of the ligase and an interactor of the ligase. These predictions are reflected in our data when we performed BioID of Ub ligases RAD18 and CHIP and compared the identified hits with those of Ub-POD (Fig. 5). We introduced two important alterations into the principle of BioID to make it selective for Ub. First, we fused the BirA WT and not BirA* to the E3 ligase. BirA WT can only efficiently biotinylate the lysine present in the AP tagged in this case to the E3 ligase of interest. Second, we attached a variant of AP-tag [(−2)AP] to the N terminus of Ub to be selectively biotinylated by BirA WT in a proximity-dependent manner in the course of substrate ubiquitination (Fig. 1, A and B). We hypothesized that Ub-POD is also sensitive to the location of the catalytic domain in the E3 ligase, because according to the catalytic intermediate complex structures (*19*, *24*, *25*, *76*) (fig. S1, A and B), fusion of BirA to the opposite end of the protein relative to the catalytic domain likely places it too far from the (−2)AP-Ub~E2. For efficient biotinylation, we fused BirA to the N terminus of RAD18, which has a RING domain at the N terminus, and to the C terminus of CHIP, which contains U-box domain at the C terminus. When we fused the tag at the noncatalytic ends of RAD18 and CHIP, we still observed higher biotinylation of cellular proteins compared to the background caused by BirA alone (fig. S7, A and B). However, we have not performed a comparison of substrates identified with different tag placements, an analysis that is required to test whether the BirA tag placement plays a significant role in the Ub-POD of E3 ligases. We predict that it is likely to be case specific. Intriguingly, we also observed that the linker between the BirA and the E3 ligase plays an important role in determining the efficiency of the Ub-POD. By increasing the linker length in the case of CHIP, we were able to increase the biotinylation (Fig. 4). Furthermore, we found that both the timing of biotin addition to the cells relative to the expression of the BirA-tagged ligase and the duration of biotin exposure can also be important parameters that can be varied, depending on the E3 ligase of interest (as is the case of CHIP; Fig. 4), to allow the detection of different sets of substrates for a given E3 ligase.

The primary condition for the success of Ub-POD for a given Ub ligase is that the ligase-mediated substrate ubiquitination should occur and be preserved until isolation in the cell line of choice and under the conditions used. Use of appropriate triggers, such as UV for RAD18, should be considered for activating ligases. For preserving ubiquitination and to prevent Ub recycling, the use of MG132 and/or PR619 or other similar agents should be considered. For substrate identification through proteomics, we primarily expressed BirA alone without the candidate ligase fusion as a control. The successful use of BirA as a control for multiple ligases here also implies that it could serve as an efficient control, in principle, for other RING or U-box E3 ligases. Where known, point mutants of ligases lacking the catalytic activity can also be used as controls. Our data indicate that for ligases known to homodimerize, activity-deficient mutants might not act as efficient controls in the Ub-POD MS experiments (fig. S2).

Several available methods can also be used to identify the substrates of Ub ligases (*6*, *7*). Although the gold-standard methods, such as diGly and GPS profiling (specific for degradative ubiquitination), are very effective in the identification of ligase substrates, they are resource intensive and require acquired expertise to use (*8*–*10*). Simpler methods similar to Ub-POD that trap the substrate of Ub ligases also exist, such as UBAIT or TULIP, which depend on the fusion of Ub to the ligase to covalently trap the ligase-substrate complex (*11*, *13*). These methods depend on the ability of the ligase to use the Ub-ligase fusion, which is quite bulky compared to Ub alone, in the Ub-cascade reaction. Any laboratory with access to basic tissue culture and a MS facility would be able to use Ub-POD. Moreover, the (−2)AP-tag on Ub is very small compared to the Ub-ligase fusion used in UBAIT and related approaches. Ub-POD also enables the isolation of potential substrates in harsh denaturing conditions that extracts proteins residing in typically insoluble cellular fractions and limits the number of false-positive hits. Last, Ub-POD provides a unique opportunity to visualize the cellular localization of ubiquitination by a ligase of interest, offering a peek into the cellular function of the ligase.

### Limitations and prospects of the current study

First, although we used three distinct AP tags for Ub’s N terminus, predominantly using (−2)AP-Ub in our experiments, we have not thoroughly compared these three AP tag variants across various Ub ligases. It is worth noting that a recent study used AP(−3)-Ub or AP^GEF^-Ub (*77*); however, a comprehensive comparison of AP tags across a wide spectrum of Ub ligases is presently absent and warrants investigation. Second, our data revealed that the inclusion of a linker GSGS between CHIP and the BirA tag enhances the biotinylation of potential cellular ubiquitination targets. This approach was explored within the framework of trial-and-error strategies to optimize Ub-POD for CHIP. While this finding suggests that the flexibility between the ligase and the BirA tag influences the success of Ub-POD, the specific nature of the linker and its optimal length may vary and necessitate individual optimization. Another important parameter in Ub-POD is the pan DUB inhibitor PR619 that we used to reduce Ub recycling. On the basis of our RAD18 and CHIP Ub-POD experiments (tables S3 and S6), although the enrichment of some substrates is improved, we are on the side of caution and infer that using PR619 may not entirely solve the Ub recycling issue in Ub-POD. Ub recycling is especially detrimental for Ub-POD experiments with long biotin exposure and ubiquitination events that are rapidly regulated through DUBs. Therefore, further work needs to be carried out toward dissecting the effects of PR619 with various ligases and also compare PR619 strategy to other Ub recycling deterring approaches. Last, in an effort to validate potential substrates for RAD18, TRAF6, and CHIP, we overexpressed the respective BirA-tagged ligases followed by streptavidin pulldown and Western blotting. Although we validated potential substrates of RAD18 and CHIP using siRNA knockdown strategies (Figs. 2E and 4E), it is imperative to validate these findings using more orthogonal techniques and elucidate the biological implications of such ubiquitination events. These aspects will be central to our future endeavors.

In the current study, we demonstrated the use of Ub-POD on two classes of E3 ligases, RING and U-box. RING-type E3 ligases comprise most of the Ub ligases in humans and the principles of Ub-POD can be applied for substrate identification of all of ~600 RING E3 ligases.

Although not part of this study, we predict that Ub-POD can be used for HECT or RBR E3 ligases, which also operate on principles of complexing with Ub before transfer of Ub to the substrate. Ub-like molecules (UBLs) such as SUMO, NEDD8, FAT10, ISG15, and UFM1 are also conjugated to proteins in a similar fashion to Ub, using a near-identical cascade of enzymatic reactions (*78*). By tagging the respective ligases with BirA and UBL with (−2)AP-tag, Ub-POD can, in principle, be adapted to other UBLs as well. In light of the demonstrated applications and possible other applications discussed here, Ub-POD likely will play an important role in future Ub research.

## MATERIALS AND METHODS

### Cell culture

Human embryonic kidney epithelial cell line 293 (HEK-293) (American Type Culture Collection CRL-1573) was cultured in Dulbecco’s modified Eagle’s medium (DMEM), supplemented with 10% (v*/*v) heat-inactivated fetal bovine serum (Gibco) within a humidified 5% CO_2_ incubator at 37°C. The cell line used in this study was free from mycoplasma contamination on the basis of PCR detection.

### Reagents and antibodies

All reagents used in this study are listed in Table 1 and reconstituted according to manufacturers’ instructions. Polyclonal and monoclonal antibodies used for this study are listed in Table 2 and were used according to the manufacturers’ recommended dilutions.

**Table 1.** List of reagents.

Reagent	Manufacturer	Catalog	Concentration and time of treatment
MG132	Calbiochem	474787	10 μM
			CHIP: 6 hours
PR619	Tocris (Biotechne)	4482	10 μM
			RAD18:4 hours
			CHIP: 4 hours
			TRAF6: 15 min
Biotin	Sigma-Aldrich	B4639	100 μM
			RAD: 18–30 hours
			CHIP: 30 hours (long) and 6 hours (short)
			TRAF6: 15 min
USP2	R&D Systems	E-504	100 nM final concentration

**Table 2.** List of antibodies.

Antibody	Catalog no.	Manufacturer
Streptavidin	926-32230	Licor
	926-68079	
HA	3724-S	Cell Signaling Technology
Tubulin	NB100-690SS	Bio-Techne Ltd.
RAD18	NB100-61063	Bio-Techne Ltd.
PCNA	sc-56	Santa Cruz Biotechnology
Biotin	31852	Pierce
Phospho NF-kB	3031	Cell Signaling Technology
Calnexin	ab22595	Abcam
POLE	GTX132100	GeneTex
Annexin V	MAB3991-SP	R&D Systems
Phospho Histone H2AX (Ser^139^)	2577S	Cell Signaling Technology
TRAF6	ab33915	Abcam
HGS	ab72053	Abcam
STAM	12434-1-AP	Proteintech (ptglab)
TOLLIP	ab187198	Abcam
TAK1	5206	Cell Signaling Technology
NEMO	HPA000426	Atlas
K63	5621 S	Cell Signaling Technology

### Cloning

To tag the catalytic end of the candidate ligases, two vectors containing WT *E. coli* biotin ligase enzyme BirA tagged with HA (HA-BirA for tagging candidate ligases with N-terminal catalytic domain and BirA-HA for tagging candidate ligases with C-terminal catalytic domain) were made, in which the candidate E3 ligases can be cloned on the basis of the position of the catalytic domain. Full-length RAD18, CHIP, and TRAF6 were incorporated into required BirA vector by sequence- and ligation-independent cloning. A GSGS linker was placed in between the catalytic end of candidate ligases and BirA by site-directed mutagenesis (SDM). Mutant ligase constructs (either catalytic cysteine mutants or E2-E3 interaction interface mutants) were made also by SDM. For BioID, the arginine at 118 of BirA was mutated to glycine by SDM.

Full-length Ub was tagged at its N terminus with an Avi tag (AP-Ub). To make truncated AP-Ub constructs (−2)AP-Ub and AP(−3)-Ub, two and three amino acids from N terminus and C terminus of the Avi tag were truncated, respectively. For the (−2)AP-UbΔGG construct, glycine at 75 of Ub was replaced with a stop codon by SDM. The list of constructs used in this study are presented in Table 3.

**Table 3.** List of constructs.

HA-BirA
HA-BirAR118G
BirA-HA
BirAR118G-HA
HA-BirA-RAD18
HA-BirA-RAD18-C64D
HA-BirAR118G-RAD18
CHIP-BirA-HA
CHIP-GSGS-BirA-HA
CHIP(I235DF237A)-BirA-HA
CHIP(I235DF237A)-GSGS-BirA-HA
CHIP-GSGS-BirAR118G-HA
HA-BirA-TRAF6
HA-BirA-TRAF6-F118A
HA-BirA-TRAF6-C70A
AP-Ub
(−2)AP-Ub
AP(−3)-Ub
(−2) AP-UbΔGG

### Transient transfection

All plasmids were transfected in HEK-293 cells with polyethyleneimine (PEI) (Invitrogen) according to the manufacturer’s instructions. Briefly, the cells were seeded the day before transfection. Next day, equal amount of Avi-tagged Ub and BirA-tagged candidate ligase constructs were mixed with PEI in a reduced serum medium; the transfection mix was incubated at room temperature (RT) for at least 20 min before adding dropwise to the cells. The transfected cells were incubated overnight at 37°C, 5% CO_2_.

#### Transfection of siRNA

Control siRNA (sc-37007), RAD18 siRNA (sc-72142, human) and CHIP siRNA (sc-43555, human) were reverse transfected in HEK-293 cells using Lipofectamine RNAiMAX Reagent (Thermo Fisher Scientific, 13778030) according to the manufacturer’s instructions. Briefly, the desired concentration of siRNAs were diluted in reduced serum medium and added to tissue culture plates. After 5 min, the Lipofectamine RNAiMAX was added to each plate containing the diluted siRNA. The siRNA–Lipofectamine RNAiMAX complex was mixed gently and incubated for 20 min at RT. Appropriate number of HEK-293 cells were diluted in complete growth medium and added to each plate containing the siRNA–Lipofectamine RNAiMAX complex followed by incubation at 37°C in a CO_2_ incubator. Forty-eight hours after transfection, cells were treated as indicated in the figure.

### Biotin labeling and induction of target protein ubiquitination

Different strategies were taken to induce target protein ubiquitination by the candidate ligases. For RAD18, UV exposure was used as an inducer of DNA damage. For CHIP, the proteasome inhibitor, MG132 was used as an inducer. For TRAF6, no inducer was necessary, because overexpression itself resulted in dimerization and thus activation of E3 ligase activity.

#### Biotin labeling and UV exposure for RAD18

Cells were treated with biotin (100 μM, dissolved in DMEM) once during transfection. After 16 to 24 hours of transfection, the cells were washed in 1× phosphate-buffered saline (PBS) and exposed to UV (10 mJ/cm^2^) in a Stratalinker UV cross-linker oven (Stratagene). After UV exposure, PBS was replaced with biotin (100 μM) supplemented complete media, and the cells were further incubated for 6 hours at 37°C in a 5% CO_2_ incubator_._

#### Biotin labeling and MG132 treatment for CHIP

We took two different biotin labeling regimens for CHIP (i) shorter biotin exposure: Biotin treatment (Fig. 4B) was done only during the inducer (MG132) treatment, 16 to 24 hours after transfection. Briefly, the transfection media was removed and replaced with medium supplemented with 100 μM biotin (dissolved in DMEM) and 10 μM MG132 and incubated at 37°C in the 5% CO_2_ incubator for 6 hours. (ii) Longer biotin exposure: Biotin treatment was done once during transfection, as well as again during inducer (MG132) treatment. Briefly, 24 hours after seeding the cells, the media was removed and replaced with media supplemented with 100 μM biotin followed by transfection. After 24 hours of transfection, the media was removed and replaced with biotin (100 μM) as well as MG132 (10 μM) supplemented complete media and incubated at 37°C in the 5% CO_2_ incubator for times as indicated in figures.

#### Biotin labeling for TRAF6

For TRAF6, 100 μM biotin was added for 15 min before harvest.

### PR-619 treatment

Before the treatment of cells with PR-619, the cells were first treated with the inducer (UV or MG132), in the presence of 100 μM biotin. After 2 hours, 10 μM PR-619 was added to the media. Equal volume of dimethyl sulfoxide (DMSO) was added in mock-treated cells. After 4 hours, the cells were harvested. For Ub-POD-TRAF6 experiments, cells were treated for 15 min with 10 μM PR-619 in the presence of 100 μM biotin.

### Immunoblotting

After required treatments, the harvested cells were lysed in 1× cell lysis buffer [50 mM tris-HCl, pH (7.5), 150 mM NaCl, 1% Triton X-100, 0.1% SDS, benzonase (250 U/μl), 1 mM EGTA, 50 mM *N*-ethylmaleimide (NEM), and protease inhibitor cocktail (Sigma-Aldrich)], sonicated for 5 min and kept on ice (4°C) for 30 min. After pelleting the debris, the protein concentrations in the supernatant were measured using a bicinchoninic acid assay (BCA) assay (Thermo Fisher Scientific). The cell lysates were run on 4 to 20% tris glycine gradient gels (Bio-Rad) by SDS-PAGE followed by transfer to polyvinylidene difluoride (PVDF) membranes. After the transfer, the membranes were incubated in blocking buffer [5% bovine serum albumin (BSA) in 1× PBS] for 30 min at RT followed by overnight incubation with the antibodies listed in Table 2 at 4°C. Next day, the membranes were incubated with fluorescent tagged secondary antibodies (1:20000) for 45 min at RT followed by imaging on a fluorescence scanner imaging system (Bio-Rad). For horseradish peroxidase (HRP)–conjugated secondary antibodies (1:5000), membranes were incubated for 1 hour at RT followed by detection with an Immobilon Crescendo Western HRP substrate (WBLUR0100, Millipore). Imaging was done using Image lab software (v5.2.1) in ChemiDoc MP Imaging System (Bio-Rad).

### Streptavidin pulldown and immunoblotting

After required treatments, the harvested cells were lysed in 1× cell lysis buffer [50 mM tris-HCl, (pH 7.5), 150 mM NaCl, 1% Triton X-100, 0.1% SDS, benzonase (250 U/μl), 1 mM EGTA, 50 mM NEM, and protease inhibitor cocktail (Sigma-Aldrich)]. Sonication was done for 5 min followed by incubation of the lysates for 30 min at 4°C. After pelleting the debris, the protein concentrations in the supernatant were measured using a BCA assay (Thermo Fisher Scientific). The lysates (2 to 5%) were kept as input. For streptavidin pulldown, the streptavidin agarose beads (Thermo Fisher Scientific) were washed twice with the 1× cell lysis buffer and incubated with 1 mg of lysed cell extracts for 2 hours at 4°C subjected to end-to-end rotation. Streptavidin-agarose beads (40 to 60 μl) were used for pulling down 1 to 3 mg of lysed cell extracts (Figs. 2D and 4D). The beads were washed twice in wash buffer 1 [50 mM tris-HCl (pH 7.5), 500 mM NaCl, 1% SDS, 1% Triton X-100, 10 mM dithiothreitol (DTT), 1 mM EGTA, and 50 mM NEM] and twice more with wash buffer 2 [50 mM tris-HCl (pH 7.5), 150 mM NaCl, 0.1% SDS, 1% Triton X-100, 10 mM DTT, 1 mM EGTA, and 50 mM NEM]. The final wash was done in wash buffer 3 [50 mM tris-HCl (pH 7.5), 150 mM NaCl, and 50 mM NEM]. All the washing steps were done for 5 min on a rotation wheel at 4°C. Proteins were eluted by boiling with 2× gel loading dye. Input lysates were also boiled with 2× gel loading dye. For proteomics analysis subsequent to streptavidin pulldown, 10% of the elute was kept for immunoblotting, and rest was kept for proteomics analysis. For substrate validation and other experiments, the pulldown and input lysates were loaded on 4 to 20% tris glycine gradient gels (Bio-Rad). For detecting protein biotinylation, the proteins were transferred on a low-fluorescence PVDF membrane followed by incubation with fluorescent-tagged streptavidin antibody (1:5000 dilution in 1× PBS containing 5% BSA, 0.2% Tween 20, and 0.1% SDS) for 45 min at RT. The membranes were washed three times, each for 5 min, with 1× PBS containing 0.2% Tween 20, and then once more with 1× PBS. Imaging was done using Image lab software (v5.2.1) in ChemiDocMP Imaging System (Bio-Rad).

### USP2 treatment

HEK-293 cells were transiently transfected with the BirA-tagged candidate ligases and (−2)AP-Ub followed by treatment with UV (for RAD18) and MG132 (for CHIP). For RAD18, biotin treatment was done as mentioned in Table 1. For CHIP, short biotin treatment regimen was followed. The lysis and pulldown were performed under nondenaturing conditions. The cells were lysed with the lysis buffer containing 50 mM tris (pH 7.5), 150 mM NaCl, 1% Triton X-100, 100 mM NEM, and Protease Inhibitor tablet followed by sonication for 5 min in water bath. The lysates were divided in two equal halves and pulldown was performed on streptavidin beads (40 μl) for 2 hours at 4°C subjected to end-to end rotation. Protein (1 to 2 mg) was used for each pulldown. The lysates (2%) were kept as input. For USP2 treatment, streptavidin pulldown beads were washed in wash buffer without NEM [50 mM tris (pH 7.5), 150 mM NaCl, and Protease Inhibitor tablet], resuspended in DUB reaction buffer [50 mM tris (pH 7.5), 50 mM NaCl, and 5 mM DTT] and incubated with recombinant human USP2 catalytic domain protein (E-504, R&D Systems) (final concentration 100 nM) for 30 min at 37°C. USP2-untreated beads were washed, resuspended in DUB reaction buffer, and incubated similarly. The beads were flicked occasionally to ensure mixing. After 30 min, both USP2-treated and untreated beads were washed three times with wash buffer without NEM. Elution was done by boiling the beads with 4× laemlli buffer at 95°C for 10 min.

### Immunostaining, confocal microscopy, and statistical analysis

HEK-293 cells were seeded on 22-mm coverslips coated with poly d-lysine in a six-well plate. The cells were cotransfected with HA-BirA/HA-BirA-RAD18 and (−2)AP-Ub constructs in the presence of biotin (100 μM). After 16 hours, the cells were exposed to UV (10 mJ/cm^2^) and further incubated for 6 hours in biotin (100 μM) containing complete media. The cells were then washed five times with 1× PBS and fixed with 4% PFA for 20 min at RT. After washing three times with 1× PBS, cells were blocked and permeabilized in solution 1 (5% BSA and 0.3% Triton X-100 in 1× PBS) for 3 hours at RT. After washing once with 1× PBS, the cells were incubated overnight with anti-HA tag primary antibody (1:800) (fig. S4), anti–phospho Histone H2AX (Ser^139^) (1:400) (Fig. 2E) in solution 2 (3% BSA, 0.1% Triton X-100, and 1× PBS). Next day cells were washed three times with solution 2 and two times with 1× PBS and further incubated with streptavidin-conjugated to Irdye 680 (1:200) and Alexa Fluor 488–conjugated anti-rabbit secondary antibody (1:200) for 1 hour in the dark in a humidified 37°C incubator. The cells were then washed five times with solution 2 and once with 1× PBS before mounting with ProLong antifade mountant with 4′,6-diamidino-2-phenylindole. Imaging was done in a Leica TCS SP5 confocal microscope (63× oil-immersion objective) keeping all the parameters same throughout the imaging process.

For statistical analysis, total number of transfected cells and the number of transfected cells with UV-irradiation induced RAD18 foci were counted from six different fields for each condition from two biological replicates. Percentage difference of transfected cells with UV irradiation–induced RAD18 foci between HA-BirA-RAD18 and HA-BirA counterparts were calculated using GraphPad Prism 9. Statistical significance of data were calculated by an unpaired *t* test and is marked as **** for *P* < 0.0001.

To determine colocalization between Streptavidin (red) and HA-tag (green) images were analyzed in FIJI. Colocalization analysis was parallelly done using Coloc2 plugin and JACoP plugin of ImageJ. The Pearson’s correlation coefficient was calculated on a cell-by-cell basis. The cells with 1 ≥ Pearson’s coefficient ≥ 0.5 were considered for statistical analysis. Results are indicative of 20 cells counted from two biological replicates; error bars indicate SD (*P* < 0.0001, calculated by unpaired *t* test).

For Fig. 2 (E and F), five different fields from two biological replicates were analyzed to find cells with colocalization of streptavidin and phospho histone H2AX (Ser^130^) signals. First, we counted the total number of transfected cells (evident from streptavidin signal) and then calculated the percentage of transfected cells showing colocalization of red (streptavidin) and pH2AX (green) as mentioned above. Colocalization analysis was done as mentioned above. The cells with 1 ≥ Pearson’s coefficient ≥ 0.5 were considered for statistical analysis, and the percentage of cells with 1 ≥ Pearson’s correlation coefficient ≥ 0.5 were plotted. For the HA-BirA-RAD18 counterpart, 50 to 90% of cells across 10 different fields from two biological replicates were found to have 1 ≥ Pearson’s correlation coefficient ≥ 0.5. In the HA-BirA control, no cells showed colocalization.

### BioID

For RAD18 and CHIP, the transfection, biotin treatment and inducer treatment regimens were followed as Ub-POD. After required treatments, cells were lysed in 1× cell lysis buffer [50 mM tris (pH 7.4), 1% Triton X-100, 150 mM NaCl, 0.4% SDS, 5 mM EDTA, 1 mM DTT, and 1× complete protease inhibitor]. The cell lysates were passed 10 to 20 times (5 to 10 strokes) through a 25-G needle, followed by sonication in a cold (4°C) water bath for 5 min. The lysates were centrifuged at 16,000*g*, 10 min, 4°C. Protein concentration was measured by BCA assay, and 2 mg of protein was used for the streptavidin pulldown. For streptavidin pulldown, 60 μl of streptavidin beads were washed by gently mixing with equilibration buffer [50 mM tris (pH 7 to 4), 150 mM NaCl, 1% Triton X-100, and 1 mM DTT]. The 2- to 3-mg lysate was incubated with the streptavidin beads for 2 hours at 4°C on a rotating wheel. After the pulldown, the beads were washed for 8 min on a rotation wheel with the following wash buffers: twice with wash buffer 1 (2% SDS in water), once with wash buffer 2 [50 mM Hepes (pH 7.4), 1 mM EDTA, 500 mM NaCl, 1% Triton X-100, and 0.1% Na-deoxycholate]; once with wash buffer 3 [10 mM tris (pH 8), 250 mM LiCl, 1 mM EDTA, 0.5% NP-40, and 0.5% Na-deoxycholate], and twice with wash buffer 4 [50 mM tris (pH 7.4), 50 mM NaCl, and 0.1% NP-40]. SDS sample buffer (2×) was added to the beads for elution followed by boiling at 95°C for 15 min. The eluted samples were used for further proteomics analysis.

### MS and data analysis for RAD18 and CHIP experiments

Reduction of disulfide bridges in cysteine containing proteins was performed with DTT [56°C, 30 min, 10 mM in 50 mM Hepes (pH 8.5)]. Reduced cysteines were alkylated with 2-chloroacetamide [room temperature, in the dark, 30 min, 20 mM in 50 mM Hepes (pH 8.5)]. Samples were prepared using the SP3 protocol (*79*, *80*), and trypsin (sequencing grade, Promega) was added in an enzyme-to-protein ratio 1:50 for overnight digestion at 37°C. Next day, peptide recovery was done in Hepes buffer by collecting supernatant on magnet and combining with second elution wash of beads with Hepes buffer. Peptides were labeled with TMT10plex (*81*) Isobaric Label Reagent (Thermo Fisher Scientific) according to the manufacturer’s instructions. Samples were combined for the TMT10plex and for further sample clean up an OASIS HLB μElution Plate (Waters) was used. Offline high-pH-reverse phase fractionation was carried out on an Agilent 1200 Infinity high-performance liquid chromatography system, equipped with a Gemini C18 column (3 μm, 110 Å, 100 by 1.0 mm, Phenomenex) (*82*).

An UltiMate 3000 RSLC nano LC system (Dionex) fitted with a trapping cartridge [μ-Precolumn C18 PepMap 100, 5 μm, 300-μm inside diameter (ID) by 5 mm, 100 Å] and an analytical column (nanoEase M/Z HSS T3 column 75 μm by 250 mm C18, 1.8 μm, 100 Å, Waters) was used. Trapping was carried out with a constant flow of trapping solution (0.05% trifluoroacetic acid in water) at 30 μl/min onto the trapping column for 6 min. Subsequently, peptides were eluted via the analytical column running solvent A (0.1% formic acid in water and 3% DMSO) with a constant flow of 0.3 μl/min, with increasing percentage of solvent B (0.1% formic acid in acetonitrile and 3% DMSO) from 2 to 8% in 6 min, then 8 to 28% for a further 66 min, in another 4 min. From 28 to 38%, this was followed by an increase of B from 38 to 80% for 3 min, and a re-equilibration back to 2% B for 5 min. The outlet of the analytical column was coupled directly to an Orbitrap Fusion Lumos Tribrid Mass Spectrometer (Thermo Fisher Scientific) using the Nanospray Flex ion source in positive-ion mode. The peptides were introduced into the Orbitrap Fusion Lumos via a Pico-Tip Emitter 360-μm OD by 20-μm ID; 10-μm tip (New Objective or CoAnn Technologies) and an applied spray voltage of 2.4 kV. The capillary temperature was set at 275°C. Full mass scan was acquired with mass range 375 to 1500 mass/charge ratio (*m*/*z*) in profile mode in the Orbitrap with resolution of 120,000. The filling time was set at maximum of 50 ms with a limitation of 4 × 10^5^ ions. Data-dependent acquisition was performed with the resolution of the Orbitrap set to 30,000, with a fill time of 94 ms and a limitation of 1 × 10^5^ ions. A normalized collision energy of 38 was applied. MS2 data were acquired in profile mode.

IsobarQuant (*83*), Mascot (v2.2.07) and MSFragger were used to process the acquired data. For MSFragger, all raw files were converted to mzmL format using MSConvert from Proteowizard59, using peak picking from the vendor algorithm. For IsobarQuant and Mascot, the acquired data were searched against Uniprot *Homo sapiens* proteome (UP000005640) containing common contaminants and reversed sequences. For MSFragger, files were searched using MSFragger v3.7 (*84*) in Fragpipe v19.1 against the Swissprot *H. sapiens* (UP000005640) database (20,594 entries) containing common contaminants and reversed sequences. The following modifications were included into the search parameters: Carbamidomethyl (C) and TMT10 (K) (fixed modification), Acetyl (Protein N-term), Oxidation (M), and TMT10 (N-term) (variable modifications). For the full scan (MS1), a mass error tolerance of 10 ppm and for MS/MS (MS2) spectra of 0.02 Da was set. Further parameters were set trypsin as protease with an allowance of maximum two missed cleavages: a minimum peptide length of seven amino acids; at least two unique peptides were required for a protein identification. The FDR on peptide and protein level was set to 0.01. GO-BP/CC/MF annotation analysis as well as GO-pathway enrichment analysis of the identified hits were performed in SRPlot (*85*).

The raw output files of IsobarQuant (protein.txt – files) or FragPipe (*84*) (protein.tsv files) were processed using the R programming language (ISBN 3-900051-07-0). Contaminants were filtered out and only proteins that were quantified with at least two unique peptides were considered for the analysis. Log_2_ transformed raw TMT reporter ion intensities were first cleaned for batch effects using limma (*86*) and further normalized using vsn (variance stabilization normalization) (*87*). Proteins were tested for differential expression using the limma package. Limma uses moderated *t* test for statistical comparison. The replicate information was added as a factor in the design matrix given as an argument to the ‘lmFit’ function of limma. A protein was annotated as a hit with an FDR smaller than 5% and an FC of at least 60% (for Ub-POD experiments) or 100% (for BioID experiments shown in Fig. 5).

### Statistical analysis for confocal images

All experiments were independently reproduced at least two times with similar results obtained. No data were excluded from analysis. Statistical analysis for confocal images was performed by blinding the investigators to group allocation. Data are presented as the means ± SD for bar graphs. *P* values were calculated using unpaired Student’s *t* test using GraphPad Prism v.9.

## METHODS FOR THE TRAF6 UB-POD

### Immunoblotting

For total lysates, cells were lysed in radioimmunoprecipitation assay (RIPA) buffer [50 mM tris, 150 mM NaCl, 0.1% SDS, 0.5% sodium deoxycholate, 1% Triton X-100, 50 μM PR-619, 1× protease inhibitors (Roche), and 1× PhosStop (Roche)] for 30 min on ice. After clearance by centrifugation at 20,000*g* for 10 min at 4°C, protein concentrations were adjusted using BCA assay. Afterwards, lysates were boiled in sample buffer [200 mM tris-HCl, 6% SDS, 20% glycerol, DTT (0.1 g/ml), and 0.01 mg Bromophenol Blue] for 5 min at 95°C. Proteins were separated by SDS-PAGE and transferred onto nitrocellulose membranes. Membranes were blocked in 5% milk or 3% BSA in TBS supplemented with 0.1% Tween 20 (TBS-T) respectively. Membranes were incubated with primary antibodies overnight at 4°C in blocking buffer, washed three times with TBS-T, incubated with secondary HRP-conjugated antibodies (1:10,000) for 1 hour at room temperature, washed again with TBS-T and immediately analyzed by enhanced chemiluminescence.

### Streptavidin pulldown and sample processing for MS

After respective treatment, cells were washed two times with ice-cold Dulbecco’s Phosphate-Buffered Saline (DPBS), scraped in PBS and pellets either processed immediately or stored at −80°C. Cells were lysed in RIPA buffer for 30 min on ice. After clearance by centrifugation at 20,000*g* for 10 min at 4°C, protein concentrations were adjusted using BCA assay. Cleared and adjusted supernatants were incubated overnight on an overhead rotator with preequilibrated streptavidin agarose (Sigma-Aldrich). Beads (45 μl) were used per each replicate experiment (10-cm dish). Next day, beads were washed two times with RIPA buffer, four times with freshly prepared 3 M Urea buffer (Urea in 50 mM ammonium bicarbonate) and suspended in a defined volume of 3 M Urea buffer. Samples were reduced with 5 mM TCEP (Sigma-Aldrich) at 55°C for 30 min, alkylated with 10 mM IAA (Sigma-Aldrich) at room temperature for 20 min and quenched with 20 mM DTT (Sigma-Aldrich). Samples were washed two times with freshly prepared 2 M Urea buffer (Urea in 50 mM ammonium bicarbonate), suspended in 50 μl of 2 M Urea buffer and digested with 1 μg of trypsin per sample at 37°C overnight. Peptides were collected by pooling the supernatant with two 50-μl 2 M Urea buffer washes, immediately acidified with 1% trifluoroacetic acid and concentrated by vacuum centrifugation. Digested peptides were desalted on custom-made C18 stage tips and reconstituted in 0.1% formic acid. For immunoblotting, beads were washed 3× with RIPA buffer and boiled in sample buffer supplemented with 30 mM biotin for 10 min at 95°C.

### Streptavidin pulldown with subsequent USP2 treatment

After lysis, the cleared supernatant was split in half and subjected to Streptavidin pulldown overnight. Beads (60 μl) were used per each replicate experiment (2× 10-cm dish). Next day, beads were washed three times with RIPA buffer and one time in reaction buffer [50 mM tris–HCl (pH 8), 10 mM NaCl, 0.01% NP-40, and 0.5 mM DTT], then treated with recombinant USP2 (500 nM) in reaction buffer for 1 hour at 30°C and washed again three times with RIPA buffer. The control nontreated supernatant was treated same except for addition of USP2.

### MS data collection and analysis for TRAF6 experiments

Peptides were separated using an Easy-nLC1200 liquid chromatograph (Thermo Fisher Scientific) followed by peptide detection on a Q Exactive HF mass spectrometer (Thermo Fisher Scientific). Samples were separated on a 75 μm × 15 cm custom-made fused silica capillary packed with C18AQ resin (Reprosil-PUR 120, 1.9 μm, Dr. Maisch) with a 35-min acetonitrile gradient in 0.1% formic acid at a flow rate of 400 nl/min (5 to 38% ACN gradient for 23 min, 38 to 60% ACN gradient for 3 min, 60 to 95% ACN gradient for 2 min). Peptides were ionized using a Nanospray Flex Ion Source (Thermo Fisher Scientific). Peptides were identified in fullMS / ddMS^2^ (Top15) mode, dynamic exclusion was enabled for 20 s and identifications with an unassigned charge or charges of one or > 8 were rejected. MS1 resolution was set to 60,000 with a scan range of 300 to 1650 *m*/*z*, MS2 resolution to 15,000. Data collection was controlled by Tune/Xcalibur (Thermo Fisher Scientific). Raw data were analyzed using MaxQuant’s (version 1.6.0.1) Andromeda search engine in reversed decoy mode based on a human reference proteome (Uniprot-FASTA, UP000005640, downloaded June 2023) with an FDR of 0.01 at both peptide and protein levels. Digestion parameters were set to specific digestion with trypsin with a maximum number of two missed cleavage sites and a minimum peptide length of seven. Oxidation of methionine and amino-terminal acetylation were set as variable and carbamidomethylation of cysteine as fixed modifications. The tolerance window was set to 20 ppm (first search) and to 4.5 ppm (main search). Label-free quantification was set to a minimum ratio count of 2, re-quantification and match-between runs was selected and at least 4 biological replicates per condition were analyzed. The raw output files of MaxQuant (ProteinGroups.txt files) were processed using the R programming language (*88*) (ISBN 3-900051-07-0). Contaminants and reverse proteins were filtered out, and only proteins that were quantified with at least two unique peptides (Razor.Peptides > = 2) were considered for the analysis. Moreover, only proteins which were identified and quantified in 3 of 4 replicates for each condition were kept. A total of 1195 proteins passed the quality control filters. Log_2_ transformed raw LFQ intensities (“LFQ.intensity” columns) were first cleaned for batch effects using the “removeBatchEffect” function of the limma package (*86*) and further normalized using the “normalizeVSN” function of the limma package (VSN - variance stabilization normalization) (*87*). Missing values were imputed with the “knn” method using the “impute” function of the Msnbase package (*89*). Proteins were tested for differential expression using a moderated *t* test by applying the limma package (“lmFit” and “eBayes” functions). The replicate information was added as a factor in the design matrix given as an argument to the lmFit function of limma. Also, imputed values were given a weight of 0.01 while quantified values were given a weight of 1 in the lmFit function. A protein was annotated as a hit with an FDR smaller than 0.05 and an FC of greater than 100%.
