# Supplementary material for: A ubiquitin-specific, proximity-based labeling approach for the identification of ubiquitin ligase substrates
Source: Sci Adv. 2024 Aug 9;10(32):eadp3000. doi: 10.1126/sciadv.adp3000 (PMC11313854; doi:10.1126/sciadv.adp3000)
Supplement: Supplementary file 1 — Figs. S1 to S7 Legends for tables S1 to S12 Legend for data S1 [file sciadv.adp3000_sm.pdf]

Science Advances
AAAS

Supplementary Materials for

A ubiquitin-specific, proximity-based labeling approach for the identification of ubiquitin ligase substrates

Urbi Mukhopadhyay *et al.*

Corresponding author: Christian Behrends, Christian.Behrends@mail03.med.uni-muenchen.de;
Sagar Bhogaraju, bhogaraju@embl.fr

Sci. Adv. **10**, eadp3000 (2024)
DOI: 10.1126/sciadv.adp3000

The PDF file includes:

Figs. S1 to S7
Legends for tables S1 to S12
Legend for data S1

Other Supplementary Material for this manuscript includes the following:

Tables S1 to S12
Data S1

Fig. S1

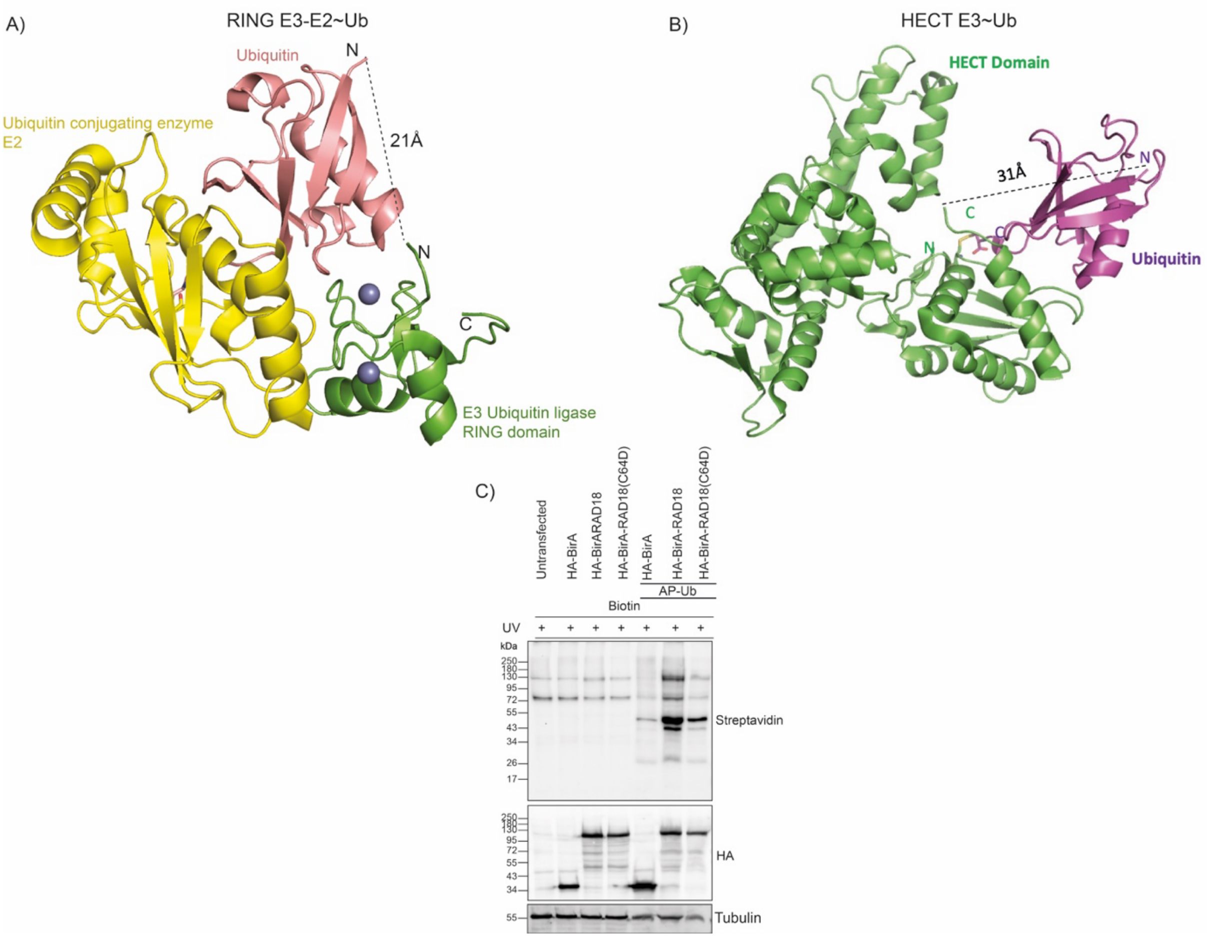

Fig. S1: **Proximity between the ubiquitin and the E3 ligase**

A) Structural model of RING E3 ligase (green) in complex with ubiquitin conjugating enzyme E2 (yellow) and ubiquitin (red). The distance between the N-terminus of Ub and the catalytic RING domain is shown.

B) Structural model of HECT-E3-ligase (green) in complex with Ub (purple) showing the distance between the catalytic HECT domain and the N-terminus of Ub.

C) HEK-293 cells transfected for 24 h with the indicated constructs were exposed to UV (10 mJ/cm^2) and allowed to recover for 6 h. Cells were kept in 100 µM biotin the whole time. Lysates were subjected to SDS-PAGE and streptavidin immunoblotting. Results are representative of two independent biological replicates.

Fig. S2

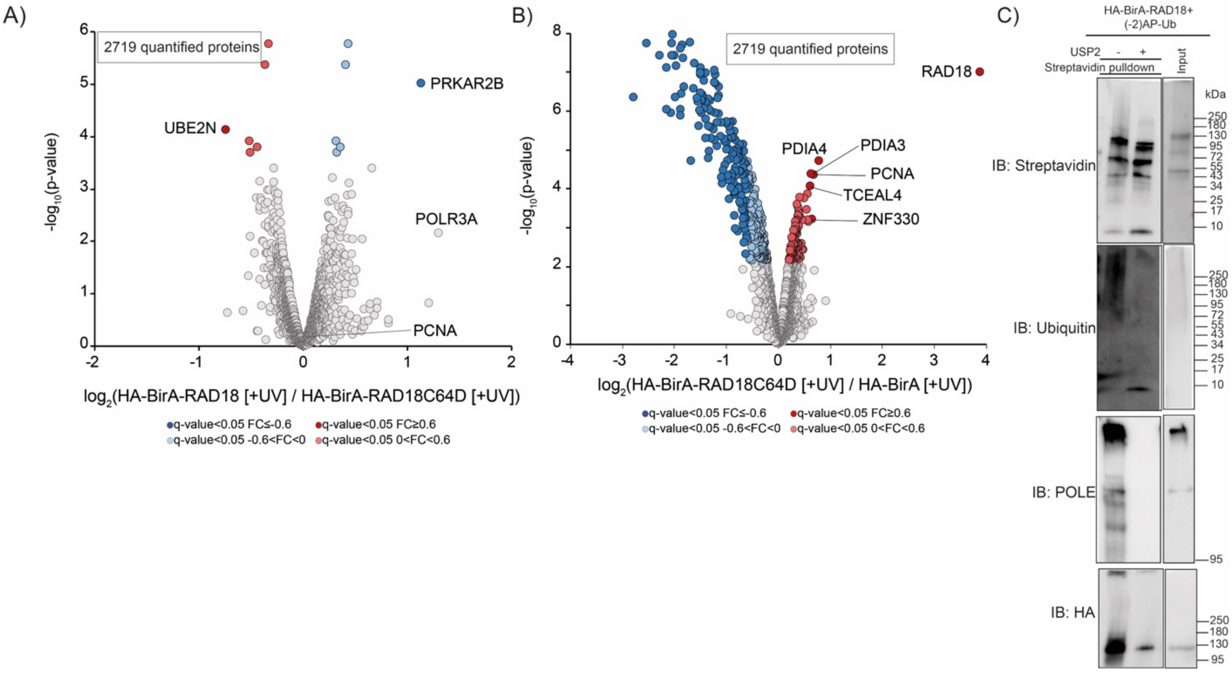

Fig. S2: Ub-POD of RAD18

A) Volcano plot depicting differentially enriched biotinylated proteins from streptavidin pulldown samples of HA-BirA-RAD18 WT and HA-BirA-RAD18 C64D expressing HEK-293 cells treated with biotin (100 µM) and UV (10 mJ/cm^2). Significantly altered proteins are labeled in dark red or blue (FDR <0.05, log2FC >I0.6I) and light red or blue (FDR <0.05, 0 > log2FC < I0.6I) (moderated t-test) (n=3 biological replicates).

B) Volcano plot of Streptavidin pulldown fraction proteins from HA-BirA-RAD18 C64D and HA-BirA expressing cells treated with biotin and UV as in Figure 2B. Significantly altered proteins are shown in dark red or blue (FDR <0.05, log2FC >I0.6I) and light red or blue (FDR <0.05, log2FC > I0.6I) (moderated t-test) (n=3 biological replicates).

C) Control for pulldown of ubiquitin-modified proteins. HEK-293 cells transiently overexpressing (-2)AP-Ub together with HA-BirA-RAD18 for 24 h. Cells were exposed to UV (10mJ/cm2) and allowed to recover for 6 hours. Biotin (100 µM) treatment was done once during transfection and again after UV exposure. Followed by streptavidin pulldown, half of this fraction was treated with deubiquitinating enzyme USP2 (500 nM) for 1 h and the other half was left untreated. Both samples were subjected to SDS-PAGE followed by immunoblotting.

Fig. S3

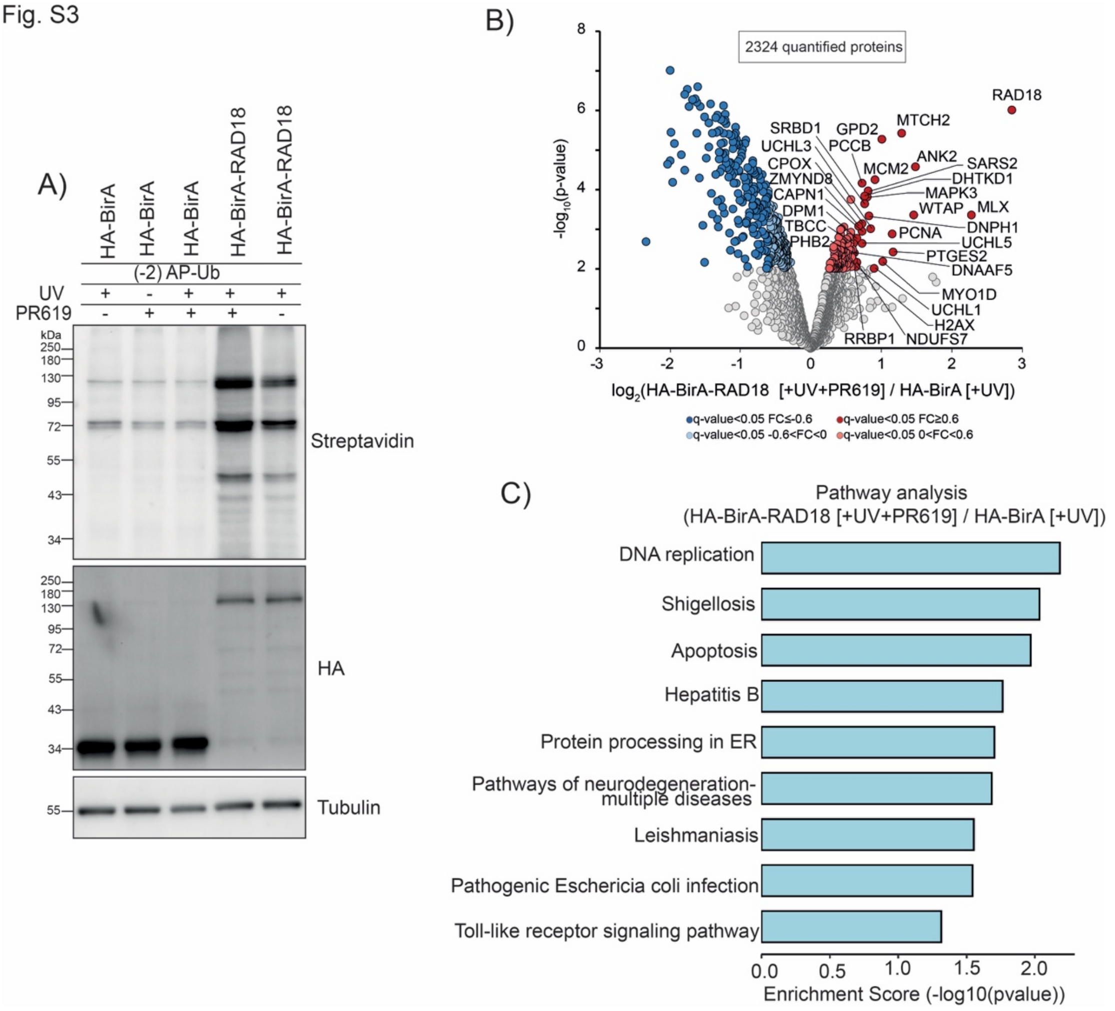

Fig. S3: Effect of the DUB inhibitor PR619 on RAD18 Ub-POD

A) HEK-293 cells transfected for 24 h with (-2)AP-Ub and HA-BirA or HA-BirA-RAD18 followed by treatment with UV (10mJ/cm2) and PR619 (10 µM) as indicated. Cells were kept in biotin (100 µM) the whole time. Lysates were subjected to SDS-PAGE and streptavidin immunoblotting.

B) MS analysis of streptavidin pulldown samples (n=3 biological replicates) of HA-BirA and HA-BirA-RAD18 transfected HEK-293 cells treated as described in A. Volcano plot of proteins labeled by HA-BirA and HA-BirA-RAD18. Significantly altered proteins are shown in dark red or blue (FDR <0.05, log2FC >I0.6I) and light red or blue (FDR <0.05, 0 > log2FC < I0.6I) (moderated t-test).

C) GO term enrichment analysis of identified hits are shown in bar graph.

Fig. S4

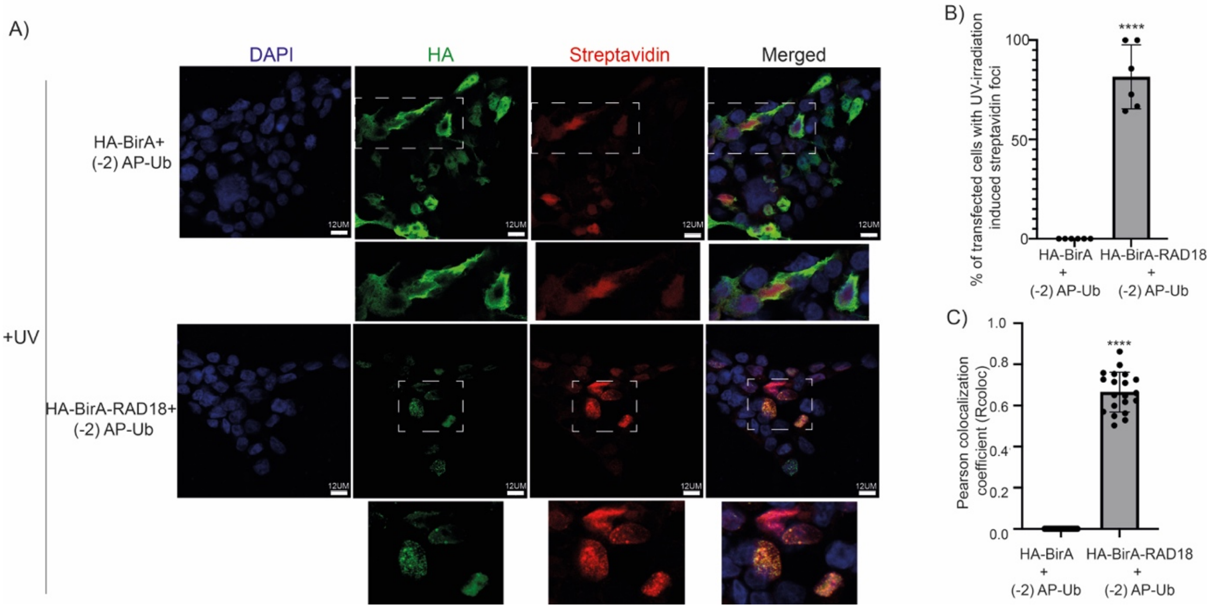

Fig. S4: Biotin localization in cells during the Ub-POD of RAD18

A) HEK-293 cells were transfected with either HA-BirA or HA-BirA-RAD18 , along with (-2)AP-Ub, treated with biotin (100 μM) and UV (10 mJ/cm^2), fixed and immunostained with anti-HA (green) and anti-streptavidin (red) antibodies. DAPI was used to stain the nucleus (blue). Scale bar 12μm.

B) Quantification of experiment shown in panel A. Total number of transfected cells were counted from (n=2 biological replicates, 6 different fields). Percentage of transfected cells with UV irradiation-induced streptavidin foci were plotted for HA-BirA and HA-BirA-RAD18 ($^{****}p<0.0001$).

C) Pearson correlation coefficient was measured to depict colocalization between red and green channels for HA-BirA and HA-BirA-RAD18 respectively. % of colocalization was plotted as a bar graph ($^{****}p<0.0001$; n=2 biological replicates, 20 cells analysed for each condition from different fields).

Fig. S5

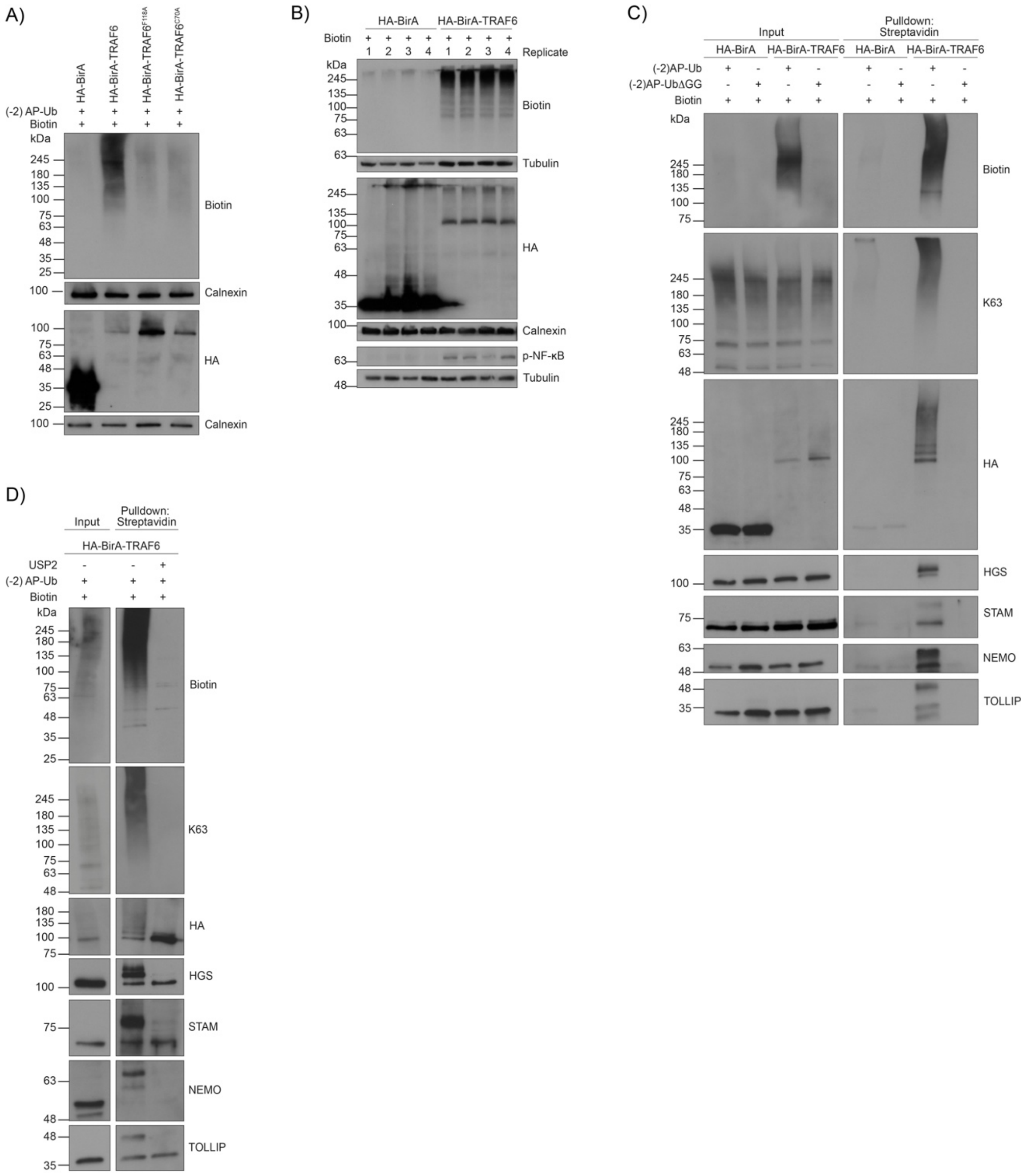

Fig. S5: Ub-POD of TRAF6

A) Control for biotinylation specificity to TRAF6 E3 ligase activity. After 24 h transfection, cells were treated with biotin (100 µM) and PR-619 (10 µM) for 15 min. Overexpression of dimerization mutant TRAF6^{F118A} as well as catalytically dead mutant TRAF6^{C70A} results in dramatic decrease of biotinylation levels in comparison to TRAF6 wt even though mutants show higher expression levels.

B) Aliquot of MS sample lysates (used for Figure 3C) are depicted to reaffirm successful biotinylation in HA-Bir-TRAF6 expressing cells, whereas HA-BirA control is showing no response to biotin treatment (n=4 independent experiments). Of note, overexpression of TRAF6 is sufficient to induce TRAF6 downstream NF-κB signaling.

C) Control for ubiquitin-dependent biotinylation. HEK-293 cells were transfected with either (-2)AP-Ub or conjugation-deficient ubiquitin mutant (-2)AP-UbDGG together with HA-BirA or HA-BirA-TRAF6 for 24 h, subjected to biotin (100 µM) and PR-619 (10 µM) for 15 min and harvested. Streptavidin pulldown clearly demonstrates the specific enrichment of biotinylated proteins only in presence of conjugatable (-2)AP-Ub and TRAF6 E3 ligase activity.

D) Control for pulldown of ubiquitin-modified proteins. HEK-293 cells transiently overexpressing (-2)AP-Ub together with HA-BirA-TRAF6 for 24 h were treated with biotin (100 µM) for 15 min and subjected to streptavidin pulldown. Half of the pulldown was treated with deubiquitinating enzyme USP2 (500 nM) for 1 h, subsequently. Pulldowns without USP2 treatment clearly show size shifted protein species, which diminish upon USP2 treatment demonstrating the pulldown of ubiquitinated proteins.

Fig. S6

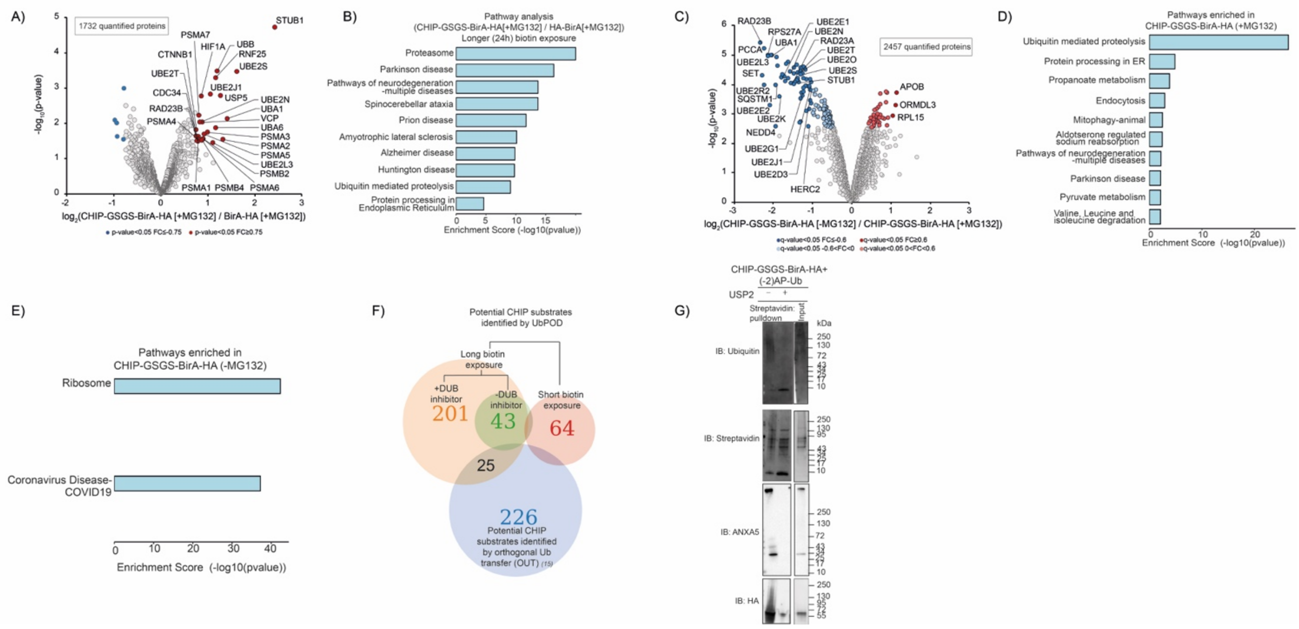

Fig. S6: Ub-POD of CHIP

A) HEK-293 cells were transfected with (-2)AP-Ub and HA-BirA or CHIP-GSGS-BirA-HA. Lysates were subjected to streptavidin pulldown and MS analysis. Volcano plot of streptavidin enriched proteins labeled by CHIP-GSGS-BirA-HA and HA-BirA in 24 h (n=3 biological replicates). Since only CHIP satisfied our FDR <0.05 criterion, we labelled potential differentially enriched proteins with p-value <0.05, log2FC >0.75 with red circles.

B) GO term analysis of enriched candidate CHIP substrates from panel A.

C) MS analysis of streptavidin pulldown samples from CHIP-GSGS-BirA-HA expressing HEK-293 cells grown in the presence or absence of MG132 (n=3 biological replicates). Volcano plot of proteins labeled by the CHIP in MG132 treated and untreated cells. Significantly altered proteins are shown in

dark red or blue (FDR <0.05, log2FC >|0.6|) and light red or blue (FDR <0.05, 0 > log2FC < |0.6|) (moderated t-test).

D and E) GO term enrichment analysis of hits identified by CHIP-GSGS-BirA-HA in the presence MG132 (D) and absence of MG132 (E). Over represented pathways are shown in bar graph.

F) Venn diagram showing overlap of identified proteins in proteomics analysis of different CHIP Ub-POD experiments and CHIP-OUT screen (*15*).

G) Control for pulldown of ubiquitin-modified proteins. HEK-293 cells were transfected with (-2)AP-Ub together with CHIP-GSGS-BirA-HA for 24 h. Cells were treated with biotin (100 µM) and MG132 (10µM) for 6 hours and lysates were subjected to streptavidin pulldown. Half of the streptavidin pulldown was treated with USP2 (500 nM) for 1 h. Subsequently, treated and untreated samples were subjected to SDS-PAGE and western blotting probed by indicated antibodies.

Fig. S7

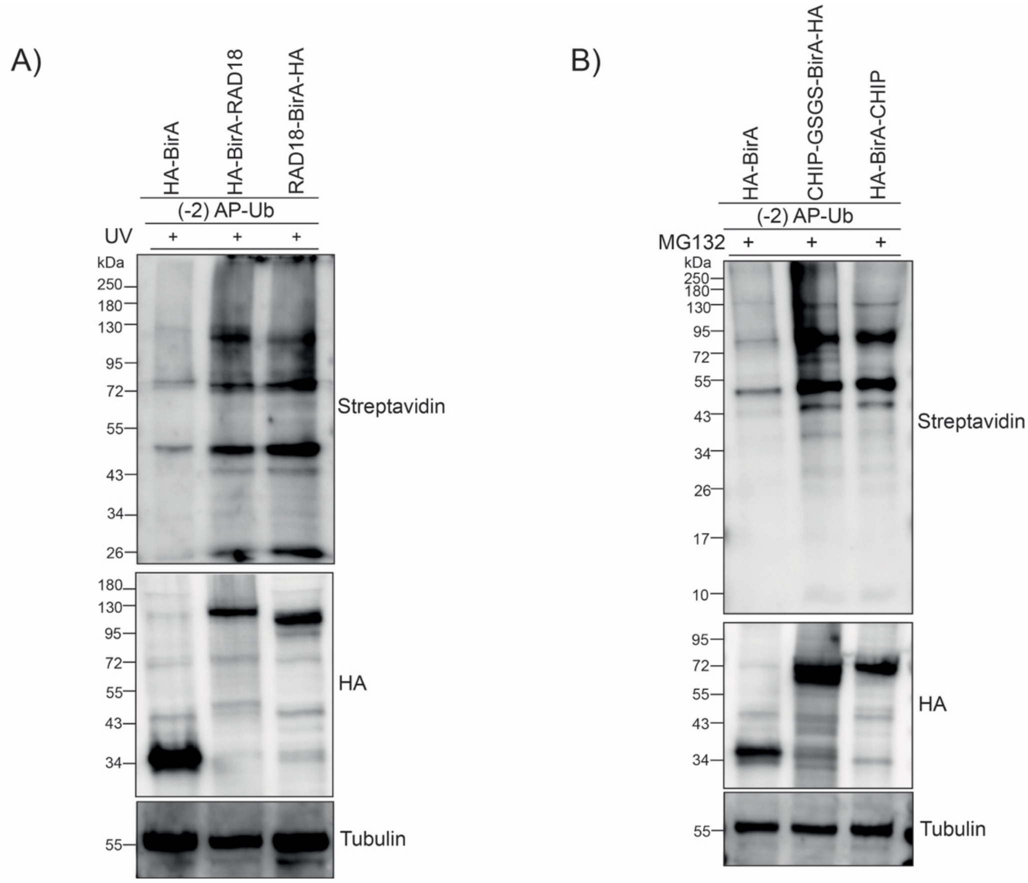

Fig. S7: Effect of BirA positioning on the Ub-POD

A) HEK-293 cells transfected with (-2)AP-Ub and HA-BirA, HA-BirA-RAD18 or RAD18-BirA-HA were incubated with biotin and exposed to UV (10mJ/cm^2) and allowed to recover for 6 h. Lysates were subjected to SDS-PAGE and streptavidin immunoblotting. Results are representative of two independent experiments.

B) HEK-293 cells transfected with (-2)AP-Ub and CHIP-GSGS-BirA-HA or HA-BirA-CHIP were incubated with biotin and MG132. Lysates were subjected to SDS-PAGE and streptavidin immunoblotting. Results are representative of two independent biological replicates.

Table S1. (separate file) Ub-POD proteomics of HA-BirA-RAD18 [+UV] versus HA-BirA-RAD18 [-UV] (Related to Fig.1D)

The “limma” tab contains data that is used for the volcano plot shown in Fig.1D. “Full dataset” tab contains additional data columns including TMT intensities. Comparison 2 tab contains data that is not used in the manuscript but was part of the same TMT experiment.

Table S2. (separate file) Ub-POD proteomics of HA-BirA-RAD18 and HA-BirA-RAD18 (C64D) mutant (Related to Fig. 2C and Fig. S2, A and B)

Three different tabs contain comparison data used for volcano plots of Ub-POD proteomics of **(Fig. 2C)** HA-BirA-RAD18 [+UV] / HA-BirA [+UV] **(Fig. S2A)** HA-BirA-RAD18 [+UV] / HA-BirA RAD18 C64D [+UV] **(Fig. S2B)** HA-BirA-RAD18 C64D [+UV] / HA-BirA [+UV]. “Full dataset” tab contains additional data columns including TMT intensities.

Table S3. (separate file) Effect of PR619 on RAD18 Ub-POD (Related to Fig. S3B)

The “limma” tab contains data that is used for the volcano plot shown in **Fig. S3B (**Ub-POD proteomics of HA-BirA-RAD18 [+UV+PR619] / HA-BirA [+UV]). “Full dataset” tab contains additional data columns including TMT intensities. Comparison 2 tab contains data that is not used in the manuscript but was part of the same TMT experiment.

Table S4. (separate file) Ub-POD proteomics of BirA-TRAF6 versus BirA (Related to Fig. 3C)

The “limma” tab contains data that is used for the volcano plot shown in **Fig. 3C.** “MaxQuant” tab contains additional data columns including LFQ intensities.

Table S5. (separate file) Significantly enriched proteins of Ub-POD experiment BirA-TRAF6 versus BirA (Related to Fig. 3C)

Significantly enriched proteins of Ub-POD experiment BirA-TRAF6 versus BirA are listed.

Table S6. (separate file) Ub-POD Proteomics of CHIP-GSGS-BirA-HA [+MG132] / HA-BirA [+MG132] (Related to Fig. S6A)

The “limma_used in S6A“ tab in the excel file contains comparison data of CHIP-GSGS-BirA-HA [+MG132] /HA-BirA [+MG132] with 24 hours of biotin exposure which is used in Fig. S6A. Other limma

comparison tabs are not used in this manuscript but are part of the same TMT experiment. "Full dataset" tab contains additional data columns including TMT intensities.

Table S7. (separate file) Ub-POD Proteomics of CHIP-GSGS-BirA-HA[+MG132] / HA-BirA[+MG132] with 6 hours of biotin exposure (Related to Fig. 4B).

The "limma_used in 4B" tab in the excel file contains comparison data of CHIP-GSGS-BirA-HA[+MG132] /HA-BirA[+MG132] with 6 hours of biotin exposure which is used in Fig. 4B. Other limma comparison tabs are not used in this manuscript but are part of the same TMT experiment. "Full dataset" tab contains additional data columns including TMT intensities.

Table S8. (separate file) Ub-POD Proteomics of CHIP-GSGS-BirA-HA[+MG132] versus CHIP-GSGS-BirA-HA[-MG132] (Related to Fig. S6C).

The "limma" tab contains data that is used for the volcano plot shown in **Fig. S6C (**Ub-POD proteomics of CHIP-GSGS-BirA-HA[+MG132] / CHIP-GSGS-BirA-HA[-MG132]). "Full dataset" tab contains additional data columns including TMT intensities.

Table S9. (separate file) Common CHIP substrates identified by CHIP Ub-POD and CHIP-OUT (15). Related to Supplementary Figure 6F.

Table S10. (separate file) BioID Proteomics of HA-BirA*RAD18 (+UV) / HA-BirA* (+UV) (Related to Fig. 5A)

The "limma used in 5A" tab in the excel file contains comparison data of HA-BirA*RAD18 (+UV) / HA-BirA* (+UV). Other limma comparison tabs are not used in this manuscript but are part of the same TMT experiment. "Full dataset" tab contains additional data columns including TMT intensities.

Table S11. (separate file) BioID proteomics of CHIP-GSGS-BirA*HA (+MG132) / HA-BirA* (+MG132) (Related to Fig. 5D).

The "limma used in 5D" tab in the excel file contains comparison data of CHIP-GSGS-BirA*HA (+MG132) / HA-BirA* (+MG132). "Full dataset" tab contains additional data columns including TMT intensities.

Table S12. (separate file) Common CHIP substrates identified by CHIP BioID and Ub-POD experiments

Comparison between BioID-RAD18 and Ub-POD-RAD18. Related to Fig. 5

Data S1: Table Index for TMT proteomics data PXD045521

This excel file contains index information that is useful for navigating the TMT mass spectrometry data deposited in the PRIDE database with the identifier PXD045521.
